# Agents, Subsystems, and the Conservation of Information

**DOI:** 10.3390/e20050358

**Published:** 2018-05-10

**Authors:** Giulio Chiribella

**Affiliations:** 1 Department of Computer Science, University of Oxford, Parks Road, Oxford OX1 3QD, UK; giulio.chiribella@cs.ox.ac.uk; 2 Canadian Institute for Advanced Research, CIFAR Program in Quantum Information Science, 661 University Ave, Toronto, ON M5G 1M1, Canada; 3 Department of Computer Science, The University of Hong Kong, Pokfulam Road, Hong Kong, China; 4 HKU Shenzhen Institute of Research and Innovation, Yuexing 2nd Rd Nanshan, Shenzhen 518057, China

**Keywords:** subsystem, agent, conservation of information, purification, group representations, commuting subalgebras

## Abstract

Dividing the world into subsystems is an important component of the scientific method. The choice of subsystems, however, is not defined *a priori*. Typically, it is dictated by experimental capabilities, which may be different for different agents. Here, we propose a way to define subsystems in general physical theories, including theories beyond quantum and classical mechanics. Our construction associates every agent *A* with a subsystem $S_{A}$, equipped with its set of states and its set of transformations. In quantum theory, this construction accommodates the notion of subsystems as factors of a tensor product, as well as the notion of subsystems associated with a subalgebra of operators. Classical systems can be interpreted as subsystems of quantum systems in different ways, by applying our construction to agents who have access to different sets of operations, including multiphase covariant channels and certain sets of free operations arising in the resource theory of quantum coherence. After illustrating the basic definitions, we restrict our attention to closed systems, that is, systems where all physical transformations act invertibly and where all states can be generated from a fixed initial state. For closed systems, we show that all the states of all subsystems admit a canonical purification. This result extends the purification principle to a broader setting, in which coherent superpositions can be interpreted as purifications of incoherent mixtures.

## 1. Introduction

The composition of systems and operations is a fundamental primitive in our modelling of the world. It has been investigated in depth in quantum information theory [1,2], and in the foundations of quantum mechanics, where composition has played a key role from the early days of Einstein–Podolski–Rosen [3] and Schroedinger [4]. At the level of frameworks, the most recent developments are the compositional frameworks of general probabilistic theories [5,6,7,8,9,10,11,12,13,14,15] and categorical quantum mechanics [16,17,18,19,20].

The mathematical structure underpinning most compositional approaches is the structure of monoidal category [18,21]. Informally, a monoidal category describes circuits, in which wires represent systems and boxes represent operations, as in the following diagram:

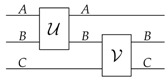
(1)

The composition of systems is described by a binary operation denoted by ⊗, and referred to as the “tensor product” (note that ⊗ is not necessarily a tensor product of vector spaces). The system A⊗B is interpreted as the composite system made of subsystems *A* and *B*. Larger systems are built in a bottom-up fashion, by combining subsystems together. For example, a quantum system of dimension $d=2^{n}$ can arise from the composition of *n* single qubits.

In some situations, having a rigid decomposition into subsystems is neither the most convenient nor the most natural approach. For example, in algebraic quantum field theory [22], it is natural to start from a single system—the field—and then to identify subsystems, e.g., spatial or temporal modes. The construction of the subsystems is rather flexible, as there is no privileged decomposition of the field into modes. Another example of flexible decomposition into subsystems arises in quantum information, where it is crucial to identify degrees of freedom that can be treated as “qubits”. Viola, Knill, and Laflamme [23] and Zanardi, Lidar, and Lloyd [24] proposed that the partition of a system into subsystems should depend on which operations are experimentally accessible. This flexible definition of subsystem has been exploited in quantum error correction, where decoherence free subsystems are used to construct logical qubits that are untouched by noise [25,26,27,28,29,30]. The logical qubits are described by “virtual subsystems" of the total Hilbert space [31], and in general such subsystems are spread over many physical qubits. In all these examples, the subsystems are constructed through an algebraic procedure, whereby the subsystems are associated with algebras of observables [32]. However, the notion of “algebra of observables” is less appealing in the context of general physical theories, because the multiplication of two observables may not be defined. For example, in the framework of general probabilistic theories [5,6,7,8,9,10,11,12,13,14,15], observables represent measurement procedures, and there is no notion of “multiplication of two measurement procedures”.

In this paper, we propose a construction of subsystems that can be applied to general physical theories, even in scenarios where observables and measurements are not included in the framework. The core of our construction is to associate subsystems to sets of *operations*, rather than observables. To fix ideas, it is helpful to think that the operations can be performed by some *agent*. Given a set of operations, the construction extracts the degrees of freedom that are acted upon *only* by those operations, identifying a “private space” that only the agent can access. Such a private space then becomes the subsystem, equipped with its own set of states and its own set of operations. This construction is closely related to an approach proposed by Krämer and del Rio, in which the states of a subsystem are identified with equivalence classes of states of the global system [33]. In this paper, we extend the equivalence relation to transformations, providing a complete description of the subsystems. We illustrate the construction in a several examples, including

1. quantum subsystems associated with the tensor product of two Hilbert spaces,
2. subsystems associated with an subalgebra of self-adjoint operators on a given Hilbert space,
3. classical systems of quantum systems,
4. subsystems associated with the action of a group representation on a given Hilbert space.

The example of the classical systems has interesting implications for the resource theory of coherence [34,35,36,37,38,39,40,41]. Our construction implies that different types of agents, corresponding to different choices of free operations, are associated with the same subsystem, namely the largest classical subsystem of a given quantum system. Specifically, classical systems arise from strictly incoherent operations [41], physically incoherent operations [38,39], phase covariant operations [38,39,40], and multiphase covariant operations (to the best of our knowledge, multiphase covariant operations have not been considered so far in the resource theory of coherence). Notably, we do not obtain classical subsystems from the maximally incoherent operations [34] and from the incoherent operations [35,36], which are the first two sets of free operations proposed in the resource theory of coherence. For these two types of operations, we find that the associated subsystem is the whole quantum system.

After examining the above examples, we explore the general features of our construction. An interesting feature is that certain properties, such as the impossibility of instantaneous signalling between two distinct subsystems, arise *by fiat*, rather then being postulated as physical requirements. This fact is potentially useful for the project of finding new axiomatizations of quantum theory [42,43,44,45,46,47,48] because it suggests that some of the axioms assumed in the usual (compositional) framework may turn out to be consequences of the very definition of subsystem. Leveraging on this fact, one could hope to find axiomatizations with a smaller number of axioms that pinpoint exactly the distinctive features of quantum theory. In addition, our construction suggests a *desideratum* that every truly fundamental axiom should arguably satisfy: *an axiom for quantum theory should hold for all possible subsystems of quantum systems*. We call this requirement *Consistency Across Subsystems.* If one accepts our broad definition of subsystems, then Consistency Across Subsystems is a very non-trivial requirement, which is not easily satisfied. For example, the Subspace Axiom [5], stating that all systems with the same number of distinguishable states are equivalent, does not satisfy Consistency Across Subsystems because classical subsystems are not equivalent to the corresponding quantum systems, even if they have the same number of distinguishable states.

In general, proving that Consistence Across Subsystems is satisfied may require great effort. Rather than inspecting the existing axioms and checking whether or not they are consistent across subsystems, one can try to formulate the axioms in a way that guarantees the validity of this property. We illustrate this idea in the case of the Purification Principle [8,12,13,15,49,50,51], which is the key ingredient in the quantum axiomatization of Refs. [13,15,42] and plays a central role in the axiomatic foundation of quantum thermodynamics [52,53,54] and quantum information protocols [8,15,55,56,57]. Specifically, we show that the Purification Principle holds for *closed systems*, defined as systems where all transformations are invertible, and where every state can be generated from a fixed initial state by the action of a suitable transformation. Closed systems satisfy the Conservation of Information [58], i.e., the requirement that physical dynamics should send distinct states to distinct states. Moreover, the states of the closed systems can be interpreted as “pure”. In this setting, the general notion of subsystem captures the idea of purification, and extends it to a broader setting, allowing us to regard coherent superpositions as the “purifications” of classical probability distributions.

The paper is structured as follows. In Section 2, we outline related works. In Section 3, we present the main framework and the construction of subsystems. The framework is illustrated with five concrete examples in Section 4. In Section 5, we discuss the key structures arising from our construction, such as the notion of partial trace and the validity of the no-signalling property. In Section 6, we identify two requirements, concerning the existence of agents with non-overlapping sets of operations, and the ability to generate all states from a given initial state. We also highlight the relation between the second requirement and the notion of causality. We then move to systems satisfying the Conservation of Information (Section 7) and we formalize an abstract notion of closed systems (Section 8). For such systems, we provide a dynamical notion of pure states, and we prove that every subsystem satisfies the Purification Principle (Section 9). A macro-example, dealing with group representations in quantum theory is provided in Section 10. Finally, the conclusions are drawn in Section 11.

## 2. Related Works

In quantum theory, the canonical route to the definition of subsystems is to consider commuting algebras of observables, associated with independent subsystems. The idea of defining independence in terms of commutation has a long tradition in quantum field theory and, more recently, quantum information theory. In algebraic quantum field theory [22], the local subsystems associated with causally disconnected regions of spacetime are described by commuting C*-algebras. A closely related approach is to associate quantum systems to von Neumann algebras, which can be characterized as double commutants [59]. In quantum error correction, decoherence free subsystems are associated with the commutant of the noise operators [28,29,31]. In this context, Viola, Knill, and Laflamme [23] and Zanardi, Lidar, and Lloyd [24] made the point that subsystems should be defined operationally, in terms of the experimentally accessible operations. The canonical approach of associating subsystems to subalgebras was further generalized by Barnum, Knill, Ortiz, and Viola [60,61], who proposed the notion of generalized entanglement, i.e., entanglement relative to a subspace of operators. Later, Barnum, Ortiz, Somma, and Viola explored this notion in the context of general probabilistic theories [62].

The above works provided a concrete model of subsystems that inspired the present work. An important difference, however, is that here we will not use the notions of observable and expectation value. In fact, we will not use any probabilistic notion, making our construction usable also in frameworks where no notion of measurement is present. This makes the construction appealingly simple, although the flip side is that more work will have to be done in order to recover the probabilistic features that are built-in in other frameworks.

More recently, del Rio, Krämer, and Renner [63] proposed a general framework for representing the knowledge of agents in general theories (see also the Ph.D. theses of del Rio [64] and Krämer [65]). Krämer and del Rio further developed the framework to address a number of questions related to locality, associating agents to monoids of operations, and introducing a relation, called *convergence through a monoid*, among states of a global system [33]. Here, we will extend this relation to transformations, and we will propose a general definition of subsystem, equipped with its set of states and its set of transformations.

Another related work is the work of Brassard and Raymond-Robichaud on no-signalling and local realism [66]. There, the authors adopt an equivalence relation on transformations, stating that two transformations are equivalent iff they can be transformed into one another through composition with a local reversible transformation. Such a relation is related to the equivalence relation on transformations considered in this paper, in the case of systems satisfying the Conservation of Information. It is interesting to observe that, notwithstanding the different scopes of Ref. [66] and this paper, the Conservation of Information plays an important role in both. Ref. [66], along with discussions with Gilles Brassard during QIP 2017 in Seattle, provided inspiration for the present paper.

## 3. Constructing Subsystems

Here, we outline the basic definitions and the construction of subsystems.

### 3.1. A Pre-Operational Framework

Our starting point is to consider a single system *S*, with a given set of states and a given set of transformations. One could think *S* to be the whole universe, or, more modestly, our “universe of discourse”, representing the fragment of the world of which we have made a mathematical model. We denote by St(S) the set of states of the system (sometimes called the “state space”), and by Transf(S) be the set of transformations the system can undergo. We assume that Transf(S) is equipped with a composition operation ∘, which maps a pair of transformations A and B into the transformation B∘A. The transformation B∘A is interpreted as the transformation occurring when B happens right before A. We also assume that there exists an identity operation $I_{S}$, satisfying the condition $A\circ I_{S}=I_{S}\circ A=A$ for every transformation A∈Transf(A). In short, we assume that the physical transformations form a *monoid*.

We do not assume any structure on the state space St(S): in particular, we do not assume that St(S) is convex. We do assume, however, is that there is an action of the monoid Transf(S) on the set St(S): given an input state ψ∈St(S) and a transformation T∈Transf(S), the action of the transformation produces the output state Tψ∈St(S).

**Example** **1** (Closed quantum systems)**.** *Let us illustrate the basic framework with a textbook example, involving a closed quantum system evolving under unitary dynamics. Here, S is a quantum system of dimension d, and the state space St(S) is the set of pure quantum states, represented as rays on the complex vector space $C^{d}$, or equivalently, as rank-one projectors. With this choice, we have*

(2) $$\begin{array}{c} \mathrm{St}\left(S\right)=\left\{|\psi \rangle \langle \psi |\;:\;|\psi \rangle \in C^{d}\;,\;\langle \psi |\psi \rangle =1\right\}\;. \end{array}$$

*The physical transformations are represented by unitary channels, i.e., by maps of the form $\left|\psi \rangle \langle \psi \right|\mapsto U|\psi \rangle \langle \psi |U^{†}$, where $U\in M_{d}\left(C\right)$ is a unitary d-by-d matrix over the complex field. In short, we have*

(3) $$\begin{array}{c} \mathrm{Transf}\left(S\right)=\left\{U\cdot U^{†}\;:\;U\in M_{d}\left(C\right)\;,\;U^{†}U=U^{†}U=I\right\}\;, \end{array}$$

*where I is the d-by-d identity matrix. The physical transformations form a monoid, with the composition operation induced by the matrix multiplication $\left(U\cdot U^{†}\right)\circ \left(V\cdot V^{†}\right):=\left(UV\right)\cdot {\left(UV\right)}^{†}$.*

**Example** **2** (Open quantum systems)**.** *Generally, a quantum system can be in a mixed state and can undergo an irreversible evolution. To account for this scenario, we must take the state space St(S) to be the set of all density matrices. For a system of dimension d, this means that the state space is*

(4) $$\begin{array}{c} \mathrm{St}\left(S\right)=\left\{\rho \in M_{d}\left(C\right)\;:\;\rho \geq 0\;\;\mathrm{Tr}\left[\rho \right]=1\right\}\;, \end{array}$$

*where $\mathrm{Tr}\left[\rho \right]=\sum_{n=1}^{d}\langle n|\rho |n\rangle$ denotes the matrix trace, and ρ≥0 means that the matrix ρ is positive semidefinite. Transf(S) is the set of all quantum channels [67], i.e., the set of all linear, completely positive, and trace-preserving maps from $M_{d}\left(C\right)$ to itself. The action of the quantum channel T on a generic state ρ can be specified through the Kraus representation [68]*

(5) $$\begin{array}{c} T\left(\rho \right)=\sum_{i=1}^{r}T_{i}\rho T_{i}^{†}\;, \end{array}$$

*where ${\left\{T_{i}\right\}}_{i=1}^{r}\subseteq M_{d}\left(C\right)$ is a set of matrices satisfying the condition $\sum_{i=1}^{r}T_{i}^{†}T_{i}=I$. The composition of two transformations T and S is given by the composition of the corresponding linear maps.*

Note that, at this stage, there is no notion of measurement in the framework. The sets St(S) and Transf(S) are meant as a model of system *S* irrespectively of anybody’s ability to measure it, or even to operate on it. For this reason, we call this layer of the framework *pre-operational*. One can think of the pre-operational framework as the arena in which agents will act. Of course, the physical description of such an arena might have been suggested by experiments done earlier on by other agents, but this fact is inessential for the scope of our paper.

### 3.2. Agents

Let us introduce agents into the picture. In our framework, an agent *A* is identified a set of transformations, denoted as Act(A;S) and interpreted as the possible actions of *A* on *S*. Since the actions must be allowed physical processes, the inclusion Act(A;S)⊆Transf(S) must hold. It is natural, but not strictly necessary, to assume that the concatenation of two actions is a valid action, and that the identity transformation is a valid action. When these assumptions are made, Act(A;S) is a monoid. Still, the construction presented in the following will hold not only for monoids, but also for generic sets Act(A;S). Hence, we adopt the following minimal definition:

**Definition** **1** (Agents)**.** *An agent A is identified by a subset Act(A;S)⊆Transf(S).*

Note that this definition captures only one aspect of agency. Other aspects—such as the ability to gather information, make decisions, and interact with other agents—are important too, but not necessary for the scope of this paper.

We also stress that the interpretation of the subset Act(A;S)⊆Transf(S) as the set of *actions of an agent* is not strictly necessary for the validity of our results. Nevertheless, the notion of “agent” here is useful because it helps explaining the rationale of our construction. The role of the agent is somehow similar to the role of a “probe charge” in classical electromagnetism. The probe charge need not exist in reality, but helps—as a conceptual tool—to give operational meaning to the magnitude and direction of the electric field.

In general, the set of actions available to agent *A* may be smaller than the set of all physical transformations on *S*. In addition, there may be other agents that act on system *S* independently of agent *A*. We define the independence of actions in the following way:

**Definition** **2.** *Agents A and B* act independently *if the order in which they act is irrelevant, namely*

(6) $$\begin{array}{c} A\circ B=B\circ A\;,\;\forall A\in \mathrm{Act}(A;S)\;,B\in \mathrm{Act}(B;S)\;. \end{array}$$

In a very primitive sense, the above relation expresses the fact that *A* and *B* act on “different degrees of freedom” of the system.

**Remark** **1** (Commutation of transformations vs. commutation of observables)**.** *Commutation conditions similar to Equation (6) are of fundamental importance in quantum field theory, where they are known under the names of “Einstein causality” [69] and “Microcausality” [70]. However, the similarity should not mislead the reader. The field theoretic conditions are expressed in terms of operator algebras. The condition is that the operators associated with independent systems commute. For example, a system localized in a certain region could be associated with the operator algebra A, and another system localized in another region could be associated with the operator algebra B. In this situation, the commutation condition reads*

(7) $$\begin{array}{c} CD=DC\;\forall C\in A,\;\forall D\in B\;. \end{array}$$

*In contrast, Equation (6) is a condition* on the transformations *, and not on the observables, which are not even described by our framework. In quantum theory, Equation (6) is a condition on the completely positive maps, and not to the elements of the algebras A and B. In Section 4, we will bridge the gap between our framework and the usual algebraic framework, focussing on the scenario where A and B are finite dimensional von Neumann algebras.*

### 3.3. Adversaries and Degradation

From the point of view of agent *A*, it is important to identify the degrees of freedom that no other agent *B* can affect. In an adversarial setting, agent *B* can be viewed as an adversary that tries to control as much of the system as possible.

**Definition** **3** (Adversary)**.** *Let A be an agent and let Act(A;S) be her set of operations. An* adversary *of A is an agent B that acts independently of A, i.e., an agent B whose set of actions satisfies*

(8) $$\begin{array}{c} \mathrm{Act}\left(B;S\right)\subseteq \mathrm{Act}{\left(A;S\right)}^{\prime }:=\left\{B\in \mathrm{Transf}(S)\;:\;B\circ A=A\circ B\;,\forall A\in \mathrm{Act}(A;S)\right\}\;. \end{array}$$

Like the agent, the adversary is a conceptual tool, which will be used to illustrate our notion of subsystem. The adversary need not be a real physical entity, localized outside the agent’s laboratory, and trying to counteract the agent’s actions. Mathematically, the adversary is just a subset of the commutant of Act(A;S). The interpretation of *B* as an “adversary” is a way to “give life to to the mathematics”, and to illustrate the rationale of our construction.

When *B* is interpreted as an adversary, we can think of his actions as a “degradation”, which compromises states and transformations. We denote the degradation relation as ${⪰}_{B}$, and write

(9) $$\begin{array}{cc} \phi {⪰}_{B}\psi \;\mathrm{iff}\; & \exists B\in \mathrm{Act}(B;S):\psi =B\;\phi \;, \end{array}$$

(10) $$\begin{array}{cc} S{⪰}_{B}T\;\mathrm{iff}\; & \exists B_{1},B_{2}\in \mathrm{Act}\left(B;S\right):\;T=B_{1}\circ S\circ B_{2} \end{array}$$

for ϕ,ψ∈St(S) or S,T∈Transf(S).

The states that can be obtained by degrading ψ will be denoted as

(11) $$\begin{array}{c} {\mathrm{Deg}}_{B}\left(\psi \right):=\left\{B\psi \;:\;B\in \mathrm{Act}(B;S)\right\}\;. \end{array}$$

The transformations that can be obtained by degrading T will be denoted as

(12) $$\begin{array}{c} {\mathrm{Deg}}_{B}\left(T\right):=\left\{B_{1}\circ T\circ B_{2}\;:\;B_{1},B_{2}\in \mathrm{Act}\left(B;S\right)\right\}. \end{array}$$

The more operations *B* can perform, the more powerful *B* will be as an adversary. The most powerful adversary compatible with the independence condition (6) is the adversary that can implement all transformations in the commutant of Act(A;S):

**Definition** **4.** *The* maximal adversary *of agent A is the agent $A^{\prime }$ that can perform the actions $\mathrm{Act}\left(A^{\prime };S\right):=\mathrm{Act}{\left(A;S\right)}^{\prime }$.*

Note that the actions of the maximal adversary are automatically a monoid, even if the set Act(A;S) is not. Indeed,

- the identity map $I_{S}$ commutes with all operations in Act(A;S), and
- if B and $B^{\prime }$ commute with every operation in Act(A;S), then also their composition $B\circ B^{\prime }$ will commute with all the operations in Act(A;S).

In the following, we will use the maximal adversary to define the subsystem associated with agent *A*.

### 3.4. The States of the Subsystem

Given an agent *A*, we think of the subsystem $S_{A}$ to be the collection of all degrees of freedom that are unaffected by the action of the maximal adversary $A^{\prime }$. Consistently with this intuitive picture, we partition the states of *S* into disjoint subsets, with the interpretation that two states are in the same subset if and only if they correspond to the same state of subsystem $S_{A}$.

We denote by $\Lambda_{\psi }$ the subset of St(S) containing the state ψ. To construct the state space of the subsystem, we adopt the following rule:

**Rule** **1.** *If the state ψ is obtained from the state ϕ through degradation, i.e., if $\psi \in {\mathrm{Deg}}_{A^{\prime }}\left(\phi \right)$, then ψ and ϕ must correspond to the same state of subsystem $S_{A}$, i.e., one must have $\Lambda_{\psi }=\Lambda_{\phi }$.*

Rule 1 imposes that all states in the set ${\mathrm{Deg}}_{A^{\prime }}\left(\psi \right)$ must be contained in the set $\Lambda_{\psi }$. Furthermore, we have the following fact:

**Proposition** **1.** *If the sets ${\mathrm{Deg}}_{A^{\prime }}\left(\phi \right)$ and ${\mathrm{Deg}}_{A^{\prime }}\left(\psi \right)$ have non-trivial intersection, then $\Lambda_{\phi }=\Lambda_{\psi }.$*

**Proof.**  By Rule 1, every element of ${\mathrm{Deg}}_{A^{\prime }}\left(\phi \right)$ is contained in $\Lambda_{\phi }$. Similarly, every element of ${\mathrm{Deg}}_{A^{\prime }}\left(\psi \right)$ is contained in $\Lambda_{\psi }$. Hence, if ${\mathrm{Deg}}_{A^{\prime }}\left(\phi \right)$ and ${\mathrm{Deg}}_{A^{\prime }}\left(\psi \right)$ have non-trivial intersection, then also $\Lambda_{\phi }$ and $\Lambda_{\psi }$ have non-trivial intersection. Since the sets $\Lambda_{\phi }$ and $\Lambda_{\psi }$ belong to a disjoint partition, we conclude that $\Lambda_{\phi }=\Lambda_{\psi }$. ☐

Generalizing the above argument, it is clear that two states ϕ and ψ must be in the same subset $\Lambda_{\phi }=\Lambda_{\psi }$ if there exists a finite sequence $\left(\psi_{1},\psi_{2},\cdots ,\psi_{n}\right)\subseteq \mathrm{St}\left(S\right)$ such that

(13) $$\begin{array}{c} \psi_{1}=\phi \;,\;\psi_{n}=\psi \;,\;\mathrm{and}\;{\mathrm{Deg}}_{A^{\prime }}\left(\psi_{i}\right)\cap {\mathrm{Deg}}_{A^{\prime }}\left(\psi_{i+1}\right)\neq \emptyset \;\forall i\in \left\{1,2,\cdots ,n-1\right\}\;. \end{array}$$

When this is the case, we write $\phi {≃}_{A}\psi$. Note that the relation $\phi {≃}_{A}\psi$ is an equivalence relation. When the relation $\phi {≃}_{A}\psi$ holds, we say that ϕ and ψ are *equivalent for agent A*. We denote the equivalence class of the state ψ by ${\left[\psi \right]}_{A}$.

By Rule 1, the whole equivalence class ${\left[\psi \right]}_{A}$ must be contained in the set $\Lambda_{\psi }$, meaning that all states in the equivalence class must correspond to the same state of subsystem $S_{A}$. Since we are not constrained by any other condition, we make the minimal choice

(14) $$\begin{array}{c} \Lambda_{\psi }:={\left[\psi \right]}_{A}\;. \end{array}$$

In summary, the state space of system $S_{A}$ is

(15) $$\begin{array}{c} \mathrm{St}\left(S_{A}\right):=\left\{{\left[\psi \right]}_{A}:\;\psi \in \mathrm{St}\left(S\right)\right\}\;. \end{array}$$

### 3.5. The Transformations of a Subsystem

The transformations of system $S_{A}$ can also be constructed through equivalence classes. Before taking equivalence classes, however, we need a candidate set of transformations that can be interpreted as acting exclusively on subsystem $S_{A}$. The largest candidate set is the set of all transformations that commute with the actions of the maximal adversary $A^{\prime }$, namely

(16) $$\begin{array}{c} \mathrm{Act}{\left(A^{\prime };S\right)}^{\prime }=\mathrm{Act}{\left(A;S\right)}^{\prime \prime }\;. \end{array}$$

In general, $\mathrm{Act}{\left(A;S\right)}^{\prime \prime }$ could be larger than Act(A;S), in agreement with the fact the set of physical transformations of system $S_{A}$ could be larger than the set of operations that agent *A* can perform. For example, agent *A* could have access only to noisy operations, while another, more technologically advanced agent could perform more accurate operations on the same subsystem.

For two transformations S and T in $\mathrm{Act}{\left(A;S\right)}^{\prime \prime }$, the degradation relation ${⪰}_{A^{\prime }}$ takes the simple form

(17) $$\begin{array}{cc} S{⪰}_{A^{\prime }}T\;\mathrm{iff}\; & \;T=B\circ S\;\mathrm{for}\;\mathrm{some}\;B\in \mathrm{Act}(A^{\prime };S)\;. \end{array}$$

As we did for the set of states, we now partition the set $\mathrm{Act}{\left(A;S\right)}^{\prime \prime }$ into disjoint subsets, with the interpretation that two transformations act in the same way on the subsystem $S_{A}$ if and only if they belong to the same subset.

Let us denote by $\Theta_{A}$ the subset containing the transformation A. To find the appropriate partition of $\mathrm{Act}{\left(A;S\right)}^{\prime \prime }$ into disjoint subsets, we adopt the following rule:

**Rule** **2.** *If the transformation $T\in \mathrm{Act}{\left(A;S\right)}^{\prime \prime }$ is obtained from the transformation $S\in \mathrm{Act}{\left(A;S\right)}^{\prime \prime }$ through degradation, i.e., if $T\in {\mathrm{Deg}}_{A^{\prime }}\left(S\right)$, then T and S must act in the same way on the subsystem $S_{A}$, i.e., they must satisfy $\Theta_{T}=\Theta_{S}$.*

Intuitively, the motivation for the above rule is that system $S_{A}$ is *defined* as the system that is not affected by the action of the adversary.

Rule 2 implies that all transformations in ${\mathrm{Deg}}_{A^{\prime }}\left(T\right)$ must be contained in $\Theta_{T}$. Moreover, we have the following:

**Proposition** **2.** *If the sets ${\mathrm{Deg}}_{A^{\prime }}\left(S\right)$ and ${\mathrm{Deg}}_{A^{\prime }}\left(T\right)$ have non-trivial intersection, then $\Theta_{S}=\Theta_{T}$.*

**Proof.**  By Rule 2, every element of ${\mathrm{Deg}}_{A^{\prime }}\left(S\right)$ is contained in $\Theta_{S}$. Similarly, every element of ${\mathrm{Deg}}_{A^{\prime }}\left(T\right)$ is contained in $\Theta_{T}$. Hence, if ${\mathrm{Deg}}_{A^{\prime }}\left(S\right)$ and ${\mathrm{Deg}}_{A^{\prime }}\left(T\right)$ have non-trivial intersection, then also $\Theta_{S}$ and $\Theta_{T}$ have non-trivial intersection. Since the sets $\Lambda_{S}$ and $\Lambda_{T}$ belong to a disjoint partition, we conclude that $\Lambda_{S}=\Lambda_{T}$. ☐

Using the above proposition, we obtain that the equality $\Theta_{T}=\Theta_{S}$ holds whenever there exists a finite sequence $\left(A_{1},A_{2},\cdots ,A_{n}\right)\subseteq \mathrm{Act}{\left(A;S\right)}^{\prime \prime }$ such that

(18) $$\begin{array}{c} A_{1}=S\;,\;A_{n}=T\;,\;\mathrm{and}\;{\mathrm{Deg}}_{A^{\prime }}\left(A_{i}\right)\cap {\mathrm{Deg}}_{A^{\prime }}\left(A_{i+1}\right)\neq \emptyset \;\forall i\in \left\{1,2,\cdots ,n-1\right\}\;. \end{array}$$

When the above relation is satisfied, we write $S{≃}_{A}T$ and we say that *S and T are equivalent for agent A*. It is immediate to check that ${≃}_{A}$ is an equivalence relation. We denote the equivalence class of the transformation $T\in \mathrm{Act}{\left(A;S\right)}^{\prime \prime }$ as ${\left[T\right]}_{A}$.

By Rule 2, all the elements of ${\left[T\right]}_{A}$ must be contained in the set $\Theta_{T}$, i.e., they should correspond to the same transformation on $S_{A}$. Again, we make the minimal choice: we stipulate that the set $\Theta_{T}$ coincides exactly with the equivalence class ${\left[T\right]}_{A}$. Hence, the transformations of subsystem $S_{A}$ are

(19) $$\begin{array}{c} \mathrm{Transf}\left(S_{A}\right):=\left\{{\left[T\right]}_{A}:\;T\in \mathrm{Act}{\left(A;S\right)}^{\prime \prime }\right\}\;. \end{array}$$

The composition of two transformations ${\left[T_{1}\right]}_{A}$ and ${\left[T_{2}\right]}_{A}$ is defined in the obvious way, namely

(20) $$\begin{array}{c} {\left[T_{1}\right]}_{A}\circ {\left[T_{2}\right]}_{A}:={\left[T_{1}\circ T_{2}\right]}_{A}\;. \end{array}$$

Similarly, the action of the transformations on the states is defined as

(21) $$\begin{array}{c} {\left[T\right]}_{A}\;{\left[\psi \right]}_{A}:={\left[T\;\psi \right]}_{A}\;. \end{array}$$

In Appendix A, we show that definitions (20) and (21) are well-posed, in the sense that their right-hand sides are independent of the choice of representatives within the equivalence classes.

**Remark** **1.** *It is important not to confuse the transformation $T\in \mathrm{Act}{\left(A;S\right)}^{\prime \prime }$ with the equivalence class ${\left[T\right]}_{A}$: the former is a transformation* on the whole system *S, while the latter is a transformation only on subsystem $S_{A}$. To keep track of the distinction, we define the* restriction *of the transformation $T\in \mathrm{Act}{\left(A;S\right)}^{\prime \prime }$ to the subsystem $S_{A}$ via the map*

(22) $$\begin{array}{c} \pi_{A}\left(T\right):={\left[T\right]}_{A}\;. \end{array}$$

**Proposition** **3.** *The restriction map $\pi_{A}:\mathrm{Act}{\left(A;S\right)}^{\prime \prime }\to \mathrm{Transf}\left(S_{A}\right)$ is a monoid homomorphism, namely $\pi_{A}\left(I_{S}\right)=I_{S_{A}}$ and $\pi_{A}\left(S\circ T\right)=\pi_{A}\left(S\right)\circ \pi_{A}\left(T\right)$ for every pair of transformations $S,T\in \mathrm{Act}{\left(A;S\right)}^{\prime \prime }$.*

**Proof.**  Immediate from the definition (20). ☐

## 4. Examples of Agents, Adversaries, and Subsystems

In this section, we illustrate the construction of subsystems in five concrete examples.

### 4.1. Tensor Product of Two Quantum Systems

Let us start from the obvious example, which will serve as a sanity check for the soundness of our construction. Let *S* be a quantum system with Hilbert space $H_{S}=H_{A}⊗H_{B}$. The states of *S* are all the density operators on the Hilbert space $H_{S}$. The space of all linear operators from $H_{S}$ to itself will be denoted as $\mathrm{Lin}(H_{S})$, so that

(23) $$\begin{array}{c} \mathrm{St}\left(S\right)=\left\{\rho \in \mathrm{Lin}\left(H_{S}\right)\;:\;\rho \geq 0,\;\mathrm{Tr}\left[\rho \right]=1\right\}\;. \end{array}$$

The transformations are all the quantum channels (linear, completely positive, and trace-preserving linear maps) from $\mathrm{Lin}(H_{S})$ to itself. We will denote the set of all channels on system *S* as Chan(S). Similarly, we will use the notation $\mathrm{Lin}(H_{A})$ [$\mathrm{Lin}(H_{B})$] for the spaces of linear operators from $H_{A}$ [$H_{B}$] to itself, and the notation Chan(A) [Chan(B)] for the quantum channels from $\mathrm{Lin}(H_{A})$ [$\mathrm{Lin}(H_{B})$] to itself.

We can now define an agent *A* whose actions are all quantum channels acting locally on system *A*, namely

(24) $$\begin{array}{c} \mathrm{Act}\left(A;S\right):=\left\{A⊗I_{B}\;:\;A\in \mathrm{Chan}\left(A\right)\right\}\;, \end{array}$$

where $I_{B}$ denotes the identity map on $\mathrm{Lin}(H_{B})$. It is relatively easy to see that the commutant of Act(A;S) is

(25) $$\begin{array}{c} \mathrm{Act}{\left(A;S\right)}^{\prime }=\left\{I_{A}⊗B\;:\;B\in \mathrm{Chan}\left(B\right)\right\} \end{array}$$

(see Appendix B for the proof). Hence, the maximal adversary of agent *A* is the adversary $A^{\prime }=B$ that has full control on the Hilbert space $H_{B}$. Note also that one has $\mathrm{Act}{\left(A;S\right)}^{\prime \prime }=\mathrm{Act}\left(A;S\right)$.

Now, the following fact holds:

**Proposition** **4.** *Two states ρ,σ∈St(S) are equivalent for agent A if and only if ${\mathrm{Tr}}_{B}\left[\rho \right]={\mathrm{Tr}}_{B}\left[\sigma \right]$, where ${\mathrm{Tr}}_{B}$ denotes the partial trace over the Hilbert space $H_{B}$.*

**Proof.**  Suppose that the equivalence $\rho {≃}_{A}\sigma$ holds. By definition, this means that there exists a finite sequence $\left(\rho_{1},\rho_{2},\cdots ,\rho_{n}\right)$ such that

(26) $$\begin{array}{c} \rho_{1}=\rho \;,\;\rho_{n}=\sigma \;,\;\mathrm{and}\;{\mathrm{Deg}}_{B}\left(\rho_{i}\right)\cap {\mathrm{Deg}}_{B}\left(\rho_{i+1}\right)\neq \emptyset \;\forall i\in \left\{1,2,\cdots ,n-1\right\}\;. \end{array}$$

In turn, the condition of non-trivial intersection implies that, for every i∈{1,2,⋯,n−1}, one has

(27) $$\begin{array}{c} \left(I_{A}⊗B_{i}\right)\;\left(\rho_{i}\right)=\left(I_{A}⊗{\tilde{B}}_{i}\right)\;\left(\rho_{i+1}\right)\;, \end{array}$$

where $B_{i}$ and ${\tilde{B}}_{i}$ are two quantum channels in Chan(B). Since $B_{i}$ and $\tilde{B_{i}}$ are trace-preserving, Equation (27) implies ${\mathrm{Tr}}_{B}\left[\rho_{i}\right]={\mathrm{Tr}}_{B}\left[\rho_{i+1}\right]$, as one can see by taking the partial trace on $H_{B}$ on both sides. In conclusion, we obtained the equality ${\mathrm{Tr}}_{B}\left[\rho \right]\equiv {\mathrm{Tr}}_{B}\left[\rho_{1}\right]={\mathrm{Tr}}_{B}\left[\rho_{2}\right]=\cdots ={\mathrm{Tr}}_{B}\left[\rho_{n}\right]\equiv {\mathrm{Tr}}_{B}\left[\sigma \right]$.

Conversely, suppose that the condition ${\mathrm{Tr}}_{B}\left[\rho \right]={\mathrm{Tr}}_{B}\left[\sigma \right]$ holds. Then, one has

(28) $$\begin{array}{c} \left(I_{A}⊗B_{0}\right)\;\left(\rho \right)=\left(I_{A}⊗B_{0}\right)\;\left(\sigma \right)\;, \end{array}$$

where $B_{0}\in \mathrm{Chan}\left(B\right)$ is the erasure channel defined as $B_{0}\left(\cdot \right)=\beta_{0}\;{\mathrm{Tr}}_{B}\left[\cdot \right]$, $\beta_{0}$ being a fixed (but otherwise arbitrary) density matrix in $\mathrm{Lin}(H_{B})$. Since $I_{A}⊗B_{0}$ is an element of Act(B;S), Equation (28) shows that the intersection between ${\mathrm{Deg}}_{B}\left(\rho \right)$ and ${\mathrm{Deg}}_{B}\left(\sigma \right)$ is non-empty. Hence, ρ and σ correspond to the same state of system $S_{A}$. ☐

We have seen that two global states ρ,σ∈St(S) are equivalent for agent *A* if and only if they have the same partial trace over *B*. Hence, the state space of the subsystem $S_{A}$ is

(29) $$\begin{array}{c} \mathrm{St}\left(S_{A}\right)=\left\{{\mathrm{Tr}}_{B}\left[\rho \right]\;:\;\rho \in \mathrm{St}\left(S\right)\right\}\;, \end{array}$$

consistently with the standard prescription of quantum mechanics.

Now, let us consider the transformations. It is not hard to show that two transformations $T,S\in \mathrm{Act}{\left(A;S\right)}^{\prime \prime }$ are equivalent if and only if ${\mathrm{Tr}}_{B}\circ T={\mathrm{Tr}}_{B}\circ S$ (see Appendix B for the details). Recalling that the transformations in $\mathrm{Act}{\left(A;S\right)}^{\prime \prime }$ are of the form $A⊗I_{B}$, for some A∈Chan(A), we obtain that the set of transformations of $S_{A}$ is

(30) $$\begin{array}{c} \mathrm{Transf}\left(S_{A}\right)=\mathrm{Chan}\left(A\right)\;. \end{array}$$

In summary, our construction correctly identifies the quantum subsystem associated with the Hilbert space $H_{A}$, with the right set of states and the right set of physical transformations.

### 4.2. Subsystems Associated with Finite Dimensional Von Neumann algebras

In this example, we show that our notion of subsystem encompasses the traditional notion of subsystem based on an algebra of observables. For simplicity, we restrict our attention to a quantum system *S* with finite dimensional Hilbert space $H_{S}≃C^{d}$, d<∞. With this choice, the state space St(S) is the set of all density matrices in $M_{d}\left(C\right)$ and the transformation monoid Transf(S) is the set of all quantum channels (linear, completely positive, trace-preserving maps) from $M_{d}\left(C\right)$ to itself.

We now define an agent *A* associated with a von Neumann algebra $A\subseteq M_{d}\left(C\right)$. In the finite dimensional setting, a von Neumann algebra is just a matrix algebra that contains the identity operator and is closed under the matrix adjoint. Every such algebra can be decomposed in a block diagonal form. Explicitly, one can decompose the Hilbert space $H_{S}$ as

(31) $$\begin{array}{c} H_{S}=\underset{k}{⨁}\;\left(H_{A_{k}}⊗H_{B_{k}}\right)\;, \end{array}$$

for appropriate Hilbert spaces $H_{A_{k}}$ and $H_{B_{k}}$. Relative to this decomposition, the elements of the algebra A are characterized as

(32) $$\begin{array}{c} C\in A\;⟺\;C=\underset{k}{⨁}\left(C_{k}⊗I_{B_{k}}\right)\;, \end{array}$$

where $C_{k}$ is an operator in $\mathrm{Lin}(H_{A_{k}})$, and $I_{B_{k}}$ is the identity on $H_{B_{k}}$. The elements of the commutant algebra $A^{\prime }$ are characterized as

(33) $$\begin{array}{c} D\in A^{\prime }\;⟺\;D=\underset{k}{⨁}\left(I_{A_{k}}⊗D_{k}\right)\;, \end{array}$$

where $I_{A_{k}}$ is the identity on $H_{A_{k}}$ and $D_{k}$ is an operator in $\mathrm{Lin}(H_{B_{k}})$.

We grant agent *A* the ability to implement all quantum channels with Kraus operators in the algebra A, i.e., all quantum channels in the set

(34) $$\begin{array}{cc} \mathrm{Chan}(A) & :=\left\{C\in \mathrm{Chan}\left(S\right)\;:\;C\left(\cdot \right)=\sum_{i=1}^{r}C_{i}\cdot C_{i}^{†}\;,\;C_{i}\in A\;\forall i\in \left\{1,\cdots ,r\right\}\right\}\;. \end{array}$$

The maximal adversary of agent *A* is the agent *B* who can implement all the quantum channels that commute with the channels in Chan(A), namely

(35) $$\begin{array}{c} \mathrm{Act}\left(B;S\right)=\mathrm{Chan}{\left(A\right)}^{\prime }\;. \end{array}$$

In Appendix C, we prove that $\mathrm{Chan}{\left(A\right)}^{\prime }$ coincides with the set of quantum channels with Kraus operators in the commutant of the algebra A: in formula,

(36) $$\begin{array}{c} \mathrm{Chan}{\left(A\right)}^{\prime }=\mathrm{Chan}\left(A^{\prime }\right)\;. \end{array}$$

As in the previous example, the states of subsystem $S_{A}$ can be characterized as “partial traces” of the states in *S*, provided that one adopts the right definition of “partial trace”. Denoting the commutant of the algebra A by $B:=A^{\prime }$, one can define the “partial trace over the algebra B” as the channel ${\mathrm{Tr}}_{B}:\mathrm{Lin}\left(H_{S}\right)\to {⨁}_{k}\mathrm{Lin}\left(H_{A_{k}}\right)$ specified by the relation

(37) $$\begin{array}{c} {\mathrm{Tr}}_{B}\left(\rho \right):=\underset{k}{⨁}\;{\mathrm{Tr}}_{B_{k}}\left[\Pi_{k}\;\rho \Pi_{k}\right]\;, \end{array}$$

where $\Pi_{k}$ is the projector on the subspace $H_{A_{k}}⊗H_{B_{k}}\subseteq H_{S}$, and ${\mathrm{Tr}}_{B_{k}}$ denotes the partial trace over the space $H_{B_{k}}$. With definition (37), is not hard to see that two states are equivalent for *A* if and only if they have the same partial trace over B:

**Proposition** **5.** *Two states ρ,σ∈St(S) are equivalent for A if and only if ${\mathrm{Tr}}_{B}\left[\rho \right]={\mathrm{Tr}}_{B}\left[\sigma \right]$.*

The proof is provided in Appendix C. In summary, the states of system $\mathrm{St}(S_{A})$ are obtained from the states of *S* via partial trace over B, namely

(38) $$\begin{array}{c} \mathrm{St}\left(S_{A}\right)=\left\{{\mathrm{Tr}}_{B}\left(\rho \right)\;:\;\rho \in \mathrm{St}\left(S\right)\right\}\;. \end{array}$$

Our construction is consistent with the standard algebraic construction, where the states of system $S_{A}$ are defined as restrictions of the global states to the subalgebra A: indeed, for every element C∈A, we have the relation

(39) $$\begin{array}{cc} \mathrm{Tr}[C\;\rho ] & =\mathrm{Tr}\left[\left(\underset{k}{⨁}\;C_{k}⊗I_{B_{k}}\right)\;\rho \right]\\ & =\sum_{k}\;\mathrm{Tr}\left[\left(C_{k}⊗I_{B_{k}}\right)\;\Pi_{k}\rho \Pi_{k}\right]\\ & =\sum_{k}\mathrm{Tr}\left\{C_{k}\;{\mathrm{Tr}}_{B_{k}}\left[\Pi_{k}\rho \Pi_{k}\right]\right\}\\ & =\mathrm{Tr}\left\{\overset{ˇ}{C}{\mathrm{Tr}}_{B}\left[\rho \right]\right\}\;,\;\overset{ˇ}{C}:=\underset{k}{⨁}C_{k}\;, \end{array}$$

meaning that the restriction of the state ρ to the subalgebra A is in one-to-one correspondence with the state ${\mathrm{Tr}}_{B}\left[\rho \right]$.

Alternatively, the states of subsystem $S_{A}$ can be characterized as density matrices of the block diagonal form

(40) $$\begin{array}{c} \sigma =\underset{k}{⨁}\;p_{k}\;\sigma_{k}\;, \end{array}$$

where $\left(p_{k}\right)$ is a probability distribution, and each $\sigma_{k}$ is a density matrix in $\mathrm{Lin}(H_{A_{k}})$. In Appendix C, we characterize the transformations of the subsystem $S_{A}$ as quantum channels A of the form

(41) $$\begin{array}{c} A=\underset{k}{⨁}\;A_{k}\;, \end{array}$$

where $A_{k}:\mathrm{Lin}\left(H_{A_{k}}\right)\to \mathrm{Lin}\left(H_{A_{k}}\right)$ is a linear, completely positive, and trace-preserving map. In summary, the subsystem $S_{A}$ is a direct sum of quantum systems.

### 4.3. Coherent Superpositions vs. Incoherent Mixtures in Closed-System Quantum Theory

We now analyze an example involving only pure states and reversible transformations. Let *S* be a single quantum system with Hilbert space $H_{S}=C^{d}\;,d<\infty$, equipped with a distinguished orthonormal basis ${\{|n\rangle \}}_{n=1}^{d}$. As the state space, we consider the set of pure quantum states: in formula,

(42) $$\begin{array}{c} \mathrm{St}\left(S\right)=\left\{|\psi \rangle \langle \psi |\;:\;|\psi \rangle \in C^{d}\;,\;\langle \psi |\psi \rangle =1\right\}\;. \end{array}$$

As the set of transformations, we consider the set of all unitary channels: in formula,

(43) $$\begin{array}{c} \mathrm{Transf}\left(S\right)=\left\{U\cdot U^{†}\;:\;U\in M_{d}\left(C\right)\;,\;U^{†}U=U^{†}U=I\right\}\;. \end{array}$$

To agent *A*, we grant the ability to implement all unitary channels corresponding to diagonal unitary matrices, i.e., matrices of the form

(44) $$\begin{array}{c} U_{\theta }=\sum_{k}\;e^{i\theta_{k}}\;|k\rangle \langle k|\;,\;\theta =\left(\theta_{1},\cdots ,\theta_{d}\right)\in {\left[0,2\pi \right)}^{\times d}\;, \end{array}$$

where each phase $\theta_{k}$ can vary independently of the other phases. In formula, the set of actions of agent *A* is

(45) $$\begin{array}{c} \mathrm{Act}\left(A;S\right)=\left\{U_{\theta }\cdot U_{\theta }^{†}\;:\;U_{\theta }\in \mathrm{Lin}\left(H_{S}\right)\;,\;U_{\theta }\;\mathrm{as}\;\mathrm{in}\;\mathrm{Equation}\;(44)\right\}. \end{array}$$

The peculiarity of this example is that the actions of the maximal adversary $A^{\prime }$ are exactly the same as the actions of *A*. It is immediate to see that Act(A;S) is included in $\mathrm{Act}(A^{\prime };S)$ because all operations of agent *A* commute. With a bit of extra work, one can see that, in fact, Act(A;S) and $\mathrm{Act}(A^{\prime };S)$ coincide.

Let us look at the subsystem associated with agent *A*. The equivalence relation among states takes a simple form:

**Proposition** **6.** *Two pure states with unit vectors $|\phi \rangle ,|\psi \rangle \in H_{S}$ are equivalent for A if and only if |ψ〉=U|ϕ〉 for some diagonal unitary matrix U.*

**Proof.**  Suppose that there exists a finite sequence $(|\psi_{1}\rangle ,|\psi_{2}\rangle ,\cdots ,|\psi_{n}\rangle )$ such that

$$\begin{array}{c} |\psi_{1}\rangle =|\phi \rangle \;,\;|\psi_{n}\rangle =|\psi \rangle \;,\;\mathrm{and}\;{\mathrm{Deg}}_{A^{\prime }}(|\psi_{i}\rangle \langle \psi_{i}|)\cap {\mathrm{Deg}}_{A^{\prime }}(|\psi_{i+1}\rangle \langle \psi_{i+1}|)\neq \emptyset \;\forall i\in \left\{1,2,\cdots ,n-1\right\}\;. \end{array}$$

This means that, for every i∈{1,⋯,n−1}, there exist two diagonal unitary matrices $U_{i}$ and ${\tilde{U}}_{i}$ such that $U_{i}|\psi_{i}\rangle ={\tilde{U}}_{i}|\psi_{i+1}\rangle$, or equivalently,

(46) $$\begin{array}{c} |\psi_{i+1}\rangle ={\tilde{U}}_{i}^{†}\;U_{i}|\psi_{i}\rangle \;. \end{array}$$

Using the above relation for all values of *i*, we obtain |ψ〉=U|ϕ〉 with $U:={\tilde{U}}_{n-1}^{†}\;U_{n-1}\cdots {\tilde{U}}_{2}^{†}\;U_{2}{\tilde{U}}_{1}^{†}\;U_{1}$.

Conversely, suppose that the condition |ψ〉=U|ϕ〉 holds for some diagonal unitary matrix *U*. Then, the intersection ${\mathrm{Deg}}_{A^{\prime }}(|\phi \rangle \langle \phi |)\cap {\mathrm{Deg}}_{A^{\prime }}(|\psi \rangle \langle \psi |)$ is non-empty, which implies that |ϕ〉〈ϕ| and |ψ〉〈ψ| are in the same equivalence class. ☐

Using Proposition 6, it is immediate to see that the equivalence class $[|\psi \rangle {\langle \psi |]}_{A^{\prime }}$ is uniquely identified by the diagonal density matrix $\rho =\sum_{k}\;|\psi_{k}{|}^{2}\;|k\rangle \langle k|$. Hence, the state space of system $S_{A}$ is the set of diagonal density matrices

(47) $$\begin{array}{c} \mathrm{St}\left(S_{A}\right)=\left\{\rho =\sum_{k}\;p_{k}\;|k\rangle \langle k|\;:\;p_{k}\geq 0\;\forall k\;,\;\sum_{k}p_{k}=1\right\}\;. \end{array}$$

The set of transformations of system $S_{A}$ is trivial because the actions of *A* coincide with the actions of the adversary $A^{\prime }$, and therefore they are all in the equivalence class of the identity transformation. In formula, one has

(48) $$\begin{array}{c} \mathrm{Transf}\left(S_{A}\right)=\left\{I_{S_{A}}\right\}\;. \end{array}$$

### 4.4. Classical Subsystems in Open-System Quantum Theory

This example is of the same flavour as the previous one but is more elaborate and more interesting. Again, we consider a quantum system *S* with Hilbert space $H=C^{d}$. Now, we take St(S) to be the whole set of density matrices in $M_{d}\left(C\right)$ and Transf(S) to be the whole set of quantum channels from $M_{d}\left(C\right)$ to itself.

We grant to agent *A* the ability to perform every multiphase covariant channel, that is, every quantum channel M satisfying the condition

(49) $$\begin{array}{c} U_{\theta }\circ M=M\circ U_{\theta }\;\forall \theta =\left(\theta_{1},\theta_{2},\cdots ,\theta_{d}\right)\in {\left[0,2\pi \right)}^{\times d}\;, \end{array}$$

where $U_{\theta }=U_{\theta }\cdot U_{\theta }^{†}$ is the unitary channel corresponding to the diagonal unitary $U_{\theta }=\sum_{k}\;e^{i\theta_{k}}\;|k\rangle \langle k|$. Physically, we can interpret the restriction to multiphase covariant channels as the lack of a reference for the definition of the phases in the basis {|k〉,k=1,⋯,d}.

It turns out that the maximal adversary of agent *A* is the agent $A^{\prime }$ that can perform every *basis-preserving channel* B, that is, every channel satisfying the condition

(50) $$\begin{array}{c} B(|k\rangle \langle k|)=|k\rangle \langle k|\;\forall k\in \{1,\cdots ,d\}\;. \end{array}$$

Indeed, we have the following:

**Theorem** **1.** *The monoid of multiphase covariant channels and the monoid of basis-preserving channels are the commutant of one another.*

The proof, presented in Appendix D.1, is based on the characterization of the basis-preserving channels provided in [71,72].

We now show that states of system $S_{A}$ can be characterized as classical probability distributions.

**Proposition** **7.** *For every pair of states ρ,σ∈St(S), the following are equivalent:*

*1.*  *ρ and σ are equivalent for agent A,*
*2.*  *D(ρ)=D(σ), where D is the completely dephasing channel $D\left(\cdot \right):=\sum_{k}\;|k\rangle \langle k|\cdot \left|k\rangle \langle k\right|$.*

**Proof.**  Suppose that Condition 1 holds, meaning that there exists a sequence $\left(\rho_{1},\rho_{2},\cdots ,\rho_{n}\right)$ such that

(51) $$\begin{array}{c} \rho_{1}=\rho \;,\;\rho_{n}=\sigma \;,\;\forall i\in \left\{1,\cdots ,n-1\right\}\;\exists B_{i}\;,\tilde{B_{i}}\in \mathrm{Act}\left(B;S\right):\;B_{i}\left(\rho_{i}\right)=\tilde{B_{i}}\left(\rho_{i+1}\right)\;, \end{array}$$

where $B_{i}$ and $\tilde{B_{i}}$ are basis-preserving channels. The above equation implies

(52) $$\begin{array}{c} \langle k|B_{i}\left(\rho_{i}\right)|k\rangle =\langle k|\tilde{B_{i}}\left(\rho_{i+1}\right)|k\rangle \;. \end{array}$$

Now, the relation 〈k|B(ρ)|k〉=〈k|ρ|k〉 is valid for every basis-preserving channel B and for every state ρ [71]. Applying this relation on both sides of Equation (52), we obtain the condition

(53) $$\begin{array}{c} \langle k|\rho_{i}|k\rangle =\langle k|\rho_{i+1}|k\rangle \;, \end{array}$$

valid for every k∈{1,⋯,d}. Hence, all the density matrices $\left(\rho_{1},\rho_{2},\cdots ,\rho_{n}\right)$ must have the same diagonal entries, and, in particular, Condition 2 must hold.

Conversely, suppose that Condition 2 holds. Since the dephasing channel D is obviously basis-preserving, we obtained the condition ${\mathrm{Deg}}_{A^{\prime }}\left(\rho \right)\cap {\mathrm{Deg}}_{A^{\prime }}\left(\sigma \right)\neq \emptyset$, which implies that ρ and σ are equivalent for agent *A*. In conclusion, Condition 1 holds. ☐

Proposition 7 guarantees that the states of system $S_{A}$ is in one-to-one correspondence with diagonal density matrices, and therefore, with classical probability distributions: in formula,

(54) $$\begin{array}{c} \mathrm{St}\left(S_{A}\right)=\left\{{\left(p_{k}\right)}_{k=1}^{d}\;:\;p_{k}\geq 0\;\forall k\;,\;\sum_{k}p_{k}=1\;\right\}\;. \end{array}$$

The transformations of system $S_{A}$ can be characterized as *transition matrices*, namely

(55) $$\begin{array}{c} \mathrm{Transf}\left(S_{A}\right)=\left\{{\left[P_{jk}\right]}_{j\leq d,\;k\leq d}\;:\;P_{jk}\geq 0\;\forall j,k\in \left\{1,\cdots ,d\right\}\;,\;\sum_{j}\;P_{jk}=1\;\forall k\in \left\{1,\cdots ,d\right\}\right\}\;. \end{array}$$

The proof of Equation (55) is provided in Appendix D.2.

In summary, agent *A* has control on a classical system, whose states are probability distributions, and whose transformations are classical transition matrices.

### 4.5. Classical Systems From Free Operations in the Resource Theory of Coherence

In the previous example, we have seen that classical systems arise from agents who have access to the monoid of multiphase covariant channels. In fact, classical systems can arise in many other ways, corresponding to agents who have access to different monoids of operations. In particular, we find that several types of free operations in the resource theory of coherence [34,35,36,37,38,39,40,41] identify classical systems. Specifically, consider the monoids of

1. *Strictly incoherent operations* [41], i.e., quantum channels T with the property that, for every Kraus operator $T_{i}$, the map $T_{i}\left(\cdot \right)=T_{i}\cdot T_{i}$ satisfies the condition $D\circ T_{i}=T_{i}\circ D$, where D is the completely dephasing channel.
2. *Dephasing covariant operations* [38,39,40], i.e., quantum channels T satisfying the condition D∘T=T∘D.
3. *Phase covariant channels [40],* i.e., quantum channels T satisfying the condition $T\circ U_{\varphi }=U_{\varphi }\circ T$, ∀φ∈[0,2π), where $U_{\varphi }$ is the unitary channel associated with the unitary matrix $U_{\varphi }=\sum_{k}\;e^{ik\varphi }\;|k\rangle \langle k|$.
4. *Physically incoherent operations* [38,39], i.e., quantum channels that are convex combinations of channels T admitting a Kraus representation where each Kraus operator $T_{i}$ is of the form
  (56) $$\begin{array}{c} T_{i}=U_{\pi_{i}}\;U_{\theta_{i}}\;P_{i}\;, \end{array}$$
  where $U_{\pi_{i}}$ is a unitary that permutes the elements of the computational basis, $U_{\theta_{i}}$ is a diagonal unitary, and $P_{i}$ is a projector on a subspace spanned by a subset of vectors in the computational basis.

For each of the monoids 1–4, our construction yields the classical subsystem consisting of diagonal density matrices. The transformations of the subsystem are just the classical channels. The proof is presented in Appendix E.1.

Notably, other choices of free operations, such as the *maximally incoherent operations [34]* and the *incoherent operations* [35], do *not* identify classical subsystems. The maximally incoherent operations are the quantum channels T that map diagonal density matrices to diagonal density matrices, namely T∘D=D∘T∘D, where D is the completely dephasing channel. The incoherent operations are the quantum channels T with the property that, for every Kraus operator $T_{i}$, the map $T_{i}\left(\cdot \right)=T_{i}\cdot T_{i}$ sends diagonal matrices to diagonal matrices, namely $T_{i}\circ D=D\circ T_{i}\circ D$.

In Appendix E.2, we show that incoherent and maximally incoherent operations do not identify classical subsystems: the subsystem associated with these operations is the whole quantum system. This result can be understood from the analogy between these operations and non-entangling operations in the resource theory of entanglement [38,39]. Non-entangling operations do not generate entanglement, but nevertheless they cannot (in general) be implemented with local operations and classical communication. Similarly, incoherent and maximally incoherent operations do not generate coherence, but they cannot (in general) be implemented with incoherent states and coherence non-generating unitary gates. An agent that performs these operations must have access to more degrees of freedom than just a classical subsystem.

At the mathematical level, the problem is that the incoherent and maximally incoherent operations do not necessarily commute with the dephasing channel D. In our construction, commutation with the dephasing channel is essential for retrieving classical subsystems. In general, we have the following theorem:

**Theorem** **2.** *Every set of operations that*

*1.*  *contains the set of classical channels, and*
*2.*  *commutes with the dephasing channel*

*identifies a d-dimensional classical subsystem of the original d-dimensional quantum system.*

The proof is provided in Appendix E.1.

## 5. Key Structures: Partial Trace and No Signalling

In this section, we go back to the general construction of subsystems, and we analyse the main structures arising from it. First, we observe that the definition of subsystem guarantees *by fiat* the validity of the no-signalling principle, stating that operations performed on one subsystem cannot affect the state of an independent subsystem. Then, we show that our construction of subsystems allows one to build a category.

### 5.1. The Partial Trace and the No Signalling Property

We defined the states of system $S_{A}$ as equivalence classes. In more physical terms, we can regard the map $\psi \mapsto {\left[\psi \right]}_{A}$ as an operation of discarding, which takes system *S* and throws away the degrees of freedom reachable by the maximal adversary $A^{\prime }$. In our adversarial picture, “throwing away some degrees of freedom” means leaving them under the control of the adversary, and considering only the part of the system that remains under the control of the agent.

**Definition** **5.** *The* partial trace over $A^{\prime }$ *is the function ${\mathrm{Tr}}_{A^{\prime }}:\mathrm{St}\left(S\right)\to \mathrm{St}\left(S_{A}\right)$, defined by ${\mathrm{Tr}}_{A^{\prime }}\left(\psi \right)={\left[\psi \right]}_{A}$ for a generic ψ∈St(S).*

The reason for the notation ${\mathrm{Tr}}_{A^{\prime }}$ is that in quantum theory the operation ${\mathrm{Tr}}_{A^{\prime }}$ coincides with the partial trace of matrices, as shown in the example of Section 4.1. For subsystems associated with von Neumann algebras, the partial trace is the “partial trace over the algebra” defined in Section 4.2. For subsystems associated with multiphase covariant channels or dephasing covariant operations, the partial trace is the completely dephasing channel, which “traces out” the off-diagonal elements of the density matrix.

With the partial trace notation, the states of system $S_{A}$ can be succinctly written as

(57) $$\begin{array}{c} \mathrm{St}\left(S_{A}\right)=\left\{\rho ={\mathrm{Tr}}_{A^{\prime }}\left(\psi \right)\;:\;\psi \in \mathrm{St}\left(S\right)\right\}\;. \end{array}$$

Denoting $B:=A^{\prime }$, we have the important relation

(58) $$\begin{array}{cc} {\mathrm{Tr}}_{B}\circ B={\mathrm{Tr}}_{B}\; & \forall B\in \mathrm{Act}(B;S)\;. \end{array}$$

Equation (58) can be regarded as the *no signalling property*: the actions of agent *B* cannot lead to any change on the system of agent *A*. Of course, here the no signalling property holds *by fiat*, precisely because of the way the subsystems are defined!

The construction of subsystems has the merit to clarify the status of the no-signalling principle. No-signalling is often associated with space-like separation, and is heuristically justified through the idea that physical influences should propagate within the light cones. However, locality is only *a sufficient condition* for the no signalling property. Spatial separation implies no signalling, but the converse is not necessarily true: every pair of distinct quantum systems satisfies the no-signalling condition, even if the two systems are spatially contiguous. In fact, the no-signalling condition holds even for virtual subsystems of a *single*, spatially localized system. Think for example of a quantum particle localized in the xy plane. The particle can be regarded as a composite system, made of two virtual subsystems: a particle localized on the *x*-axis, and another particle localized on the *y*-axis. The no-signalling property holds for these two subsystems, even if they are not separated in space. As Equation (58) suggests, the validity of the no-signalling property has more to do with the way subsystems are constructed, rather than the way the subsystems are distributed in space.

### 5.2. A Baby Category

Our construction of subsystems defines a category, consisting of three objects, $S,S_{A}$, and $S_{B}$, where $S_{B}$ is the subsystem associated with the agent $B=A^{\prime }$. The sets Transf(S), $\mathrm{Transf}(S_{A})$, and $\mathrm{Transf}(S_{B})$ are the endomorphisms from *S* to *S*, $S_{A}$ to $S_{A}$, and $S_{B}$ to $S_{B}$, respectively. The morphisms from *S* to $S_{A}$ and from *S* to $S_{B}$ are defined as

(59) $$\begin{array}{c} \mathrm{Transf}\left(S\to S_{A}\right)=\left\{{\mathrm{Tr}}_{B}\circ T\;:\;T\in \mathrm{Transf}\left(S\right)\right\} \end{array}$$

and

(60) $$\begin{array}{c} \mathrm{Transf}\left(S\to S_{B}\right)=\left\{{\mathrm{Tr}}_{A}\circ T\;:\;T\in \mathrm{Transf}\left(S\right)\right\}\;, \end{array}$$

respectively.

Morphisms from $S_{A}$ to *S*, from $S_{B}$ to *S*, from $S_{A}$ to $S_{B}$, or from $S_{B}$ to $S_{A}$, are not naturally defined. In Appendix F, we provide a mathematical construction that enlarges the sets of transformations, making all sets non-empty. Such a construction allows us to reproduce a categorical structure known as a *splitting of idempotents* [73,74]

## 6. Non-Overlapping Agents, Causality, and the Initialization Requirement

In the previous sections, we developed a general framework, applicable to arbitrary physical systems. In this section, we identify some desirable properties that the global systems may enjoy.

### 6.1. Dual Pairs of Agents

So far, we have taken the perspective of agent *A*. Let us now take the perspective of the maximal adversary $A^{\prime }$. We consider $A^{\prime }$ as the agent, and denote his maximal adversary as $A^{\prime \prime }$. By definition, $A^{\prime \prime }$ can perform every action in the commutant of $\mathrm{Act}(A^{\prime };S)$, namely

(61) $$\begin{array}{c} \mathrm{Act}\left(A^{\prime \prime };S\right)=\mathrm{Act}{\left(A^{\prime };S\right)}^{\prime }=\mathrm{Act}{\left(A;S\right)}^{\prime \prime }\;. \end{array}$$

Obviously, the set of actions allowed to agent $A^{\prime \prime }$ includes the set of actions allowed to agent *A*. At this point, one could continue the construction and consider the maximal adversary of agent $A^{\prime \prime }$. However, no new agent would appear at this point: the maximal adversary of agent $A^{\prime \prime }$ is agent $A^{\prime }$ again. When two agents have this property, we call them a *dual pair:*

**Definition** **6.** *Two agents A and B form a dual pair iff $\mathrm{Act}\left(A;S\right)=\mathrm{Act}{\left(B;S\right)}^{\prime }$ and $\mathrm{Act}\left(B;S\right)=\mathrm{Act}{\left(A;S\right)}^{\prime }$.*

All the examples in Section 4 are examples of dual pairs of agents.

It is easy to see that an agent *A* is part of a dual pair if and only if the set Act(A;S) coincides with its double commutant $\mathrm{Act}{\left(A;S\right)}^{\prime \prime }$.

### 6.2. Non-Overlapping Agents

Suppose that agents *A* and *B* form a dual pair. In general, the actions in Act(A;S) may have a non-trivial intersection with the actions in Act(B;S). This situation does indeed happen, as we have seen in Section 4.3 and Section 4.4. Still, it is important to examine the special case where the actions of *A* and *B* have only trivial intersection, corresponding to the identity action $I_{S}$. When this is the case, we say that the agents *A* and *B* are *non-overlapping:*

**Definition** **7.** *Two agents A and B are non-overlapping iff $\mathrm{Act}\left(A;S\right)\cap \mathrm{Act}\left(B;S\right)\subseteq \left\{I_{S}\right\}$.*

Dual pairs of non-overlapping agents are characterized by the fact that the sets of actions have trivial center:

**Proposition** **8.** *Let A and B be a dual pair of agents. Then, the following are equivalent:*

*1.*  *A and B are non-overlapping,*
*2.*  *Act(A;S) has trivial center,*
*3.*  *Act(B;S) has trivial center.*

**Proof.**  Since agents *A* and *B* are dual to each other, we have $\mathrm{Act}\left(B;S\right)=\mathrm{Act}{\left(A;S\right)}^{\prime }$ and $\mathrm{Act}\left(A;S\right)=\mathrm{Act}{\left(B;S\right)}^{\prime }$. Hence, the intersection Act(A;S)∩Act(B;S) coincides with the center of Act(A;S), and with the center of Act(B;S). The non-overlap condition holds if and only if the center is trivial. ☐

Note that the existence of non-overlapping dual pairs is a condition on the transformations of the whole system *S*:

**Proposition** **9.** *The following are equivalent:*

*1.*  *system S admits a dual pair of non-overlapping agents,*
*2.*  *the monoid Transf(S) has trivial center.*

**Proof.**  Assume that Condition 1 holds for a pair of agents *A* and *B*. Let C(S) be the center of Transf(S). By definition, C(S) is contained into Act(B;S) because Act(B;S) contains all the transformations that commute with those in Act(A;S). Moreover, the elements of C(S) commute with all elements of Act(B;S), and therefore they are in the center of Act(B;S). Since *A* and *B* are a non-overlapping dual pair, the center of Act(B;S) must be trivial (Proposition 8), and therefore C(S) must be trivial. Hence, Condition 2 holds.

Conversely, suppose that Condition 2 holds. In that case, it is enough to take *A* to be the *maximal agent*, i.e., the agent $A_{\max}$ with $\mathrm{Act}\left(A_{\max};S\right)=\mathrm{Transf}\left(S\right)$. Then, the maximal adversary of $A_{\max}$ is the agent $B=A_{\max}^{\prime }$ with $\mathrm{Act}\left(B;S\right)=\mathrm{Act}{\left(A_{\max};S\right)}^{\prime }=C\left(S\right)=\left\{I_{S}\right\}$. By definition, the two agents form a non-overlapping dual pair. Hence, Condition 1 holds. ☐

The existence of dual pairs of non-overlapping agents is a desirable property, which may be used to characterize “good systems”:

**Definition** **8** (Non-Overlapping Agents)**.** *We say that system S satisfies the* Non-Overlapping Agents Requirement *if there exists at least one dual pair of non-overlapping agents acting on S.*

The Non-Overlapping Agents Requirement guarantees that the total system *S* can be regarded as a subsystem: if $A_{\max}$ is the *maximal agent* (i.e., the agent who has access to all transformations on *S*), then the subsystem $S_{A_{\max}}$ is the whole system *S*. A more formal statement of this fact is provided in Appendix G.

### 6.3. Causality

The Non-Overlapping Agents Requirement guarantees that the subsystem associated with a maximal agent (i.e., an agent who has access to all possible transformations) is the whole system *S*. On the other hand, it is natural to expect that a minimal agent, who has no access to any transformation, should be associated with the trivial system, i.e., the system with a single state and a single transformation. The fact that the minimal agent is associated with the trivial system is important because it equivalent to a property of causality [8,13,75,76]: indeed, we have the following

**Proposition** **10.** *Let $A_{\min}$ be the minimal agent and let $A_{\max}$ be its maximal adversary, coinciding with the maximal agent. Then, the following conditions are equivalent*

*1.*  *$S_{A_{\min}}$ is the trivial system,*
*2.*  *one has ${\mathrm{Tr}}_{A_{\max}}\left[\rho \right]={\mathrm{Tr}}_{A_{\max}}\left[\sigma \right]$ for every pair of states ρ,σ∈St(S).*

**Proof.**  1⇒2: By definition, the state space of $S_{A_{\min}}$ consists of states of the form ${\mathrm{Tr}}_{A_{\max}}\left[\rho \right]$, ρ∈St(S). Hence, the state space contains only one state if and only if Condition 2 holds. 2⇒1: Condition 2 implies that every two states of system *S* are equivalent for agent $A_{\max}$. The fact that $S_{A_{\min}}$ has only one transformation is true by definition: since the adversary of $A_{\min}$ is the maximal agent, one has $T\in {\mathrm{Deg}}_{A_{\max}}\left(I_{S}\right)$ for every transformation T∈Transf(S). Hence, every transformation is in the equivalence class of the identity. ☐

With a little abuse of notation, we may denote the trace over $A_{\max}$ as ${\mathrm{Tr}}_{S}$ because $A_{\max}$ has access to all transformations on system *S*. With this notation, the causality condition reads

(62) $$\begin{array}{c} {\mathrm{Tr}}_{S}\left[\rho \right]={\mathrm{Tr}}_{S}\left[\sigma \right]\;\forall \rho ,\sigma \in \mathrm{St}\left(S\right)\;. \end{array}$$

It is interesting to note that, unlike no signalling, causality does not necessarily hold in the framework of this paper. This is because the trace ${\mathrm{Tr}}_{S}$ is defined as the quotient with respect to all possible transformations, and having a single equivalence class is a non-trivial property. One possibility is to demand the validity of this property, and to call a system *proper*, only if it satisfies the causality condition (62). In the following subsection, we will see a requirement that guarantees the validity of the causality condition.

### 6.4. The Initialization Requirement

The ability to prepare states from a fixed initial state is important in the circuit model of quantum computation, where qubits are initialized to the state |0〉, and more general states are generated by applying quantum gates. More broadly, the ability to initialize the system in a given state and to generate other states from it is important for applications in quantum control and adiabatic quantum computing. Motivated by these considerations, we formulate the following definition:

**Definition** **9.** *A system S satisfies the* Initialization Requirement *if there exists a state $\psi_{0}\in \mathrm{St}\left(S\right)$ from which any other state can be generated, meaning that, for every other state ψ∈St(S), there exists a transformation T∈Transf(S) such that $\psi =T\psi_{0}$. When this is the case, the state $\psi_{0}$ is called* cyclic.

The Initialization Requirement is satisfied in quantum theory, both at the pure state level and at the mixed state level. At the pure state level, every unit vector $|\psi \rangle \in H_{S}$ can be generated from a fixed unit vector $|\psi_{0}\rangle \in H_{S}$ via a unitary transformation *U*. At the mixed state level, every density matrix ρ can be generated from a fixed density matrix $\rho_{0}$ via the erasure channel $C_{\rho }\left(\cdot \right)=\rho \;\mathrm{Tr}\left[\cdot \right]$. By the same argument, the initialization requirement is also satisfied when *S* is a system in an operational-probabilistic theory [8,10,11,12,13] and when *S* is a system in a causal process theory [75,76].

The Initialization Requirement guarantees that minimal agents are associated with trivial systems:

**Proposition** **11.** *Let S be a system satisfying the Initialization Requirement, and let $A_{\min}$ be the* minimal agent *, i.e., the agent that can only perform the identity transformation. Then, the subsystem $S_{A_{\min}}$ is trivial: $\mathrm{St}\left(S_{A_{\min}}\right)$ contains only one state and $\mathrm{Transf}\left(S_{A_{\min}}\right)$ contains only one transformation.*

**Proof.**  By definition, the maximal adversary of $A_{\min}$ is the maximal agent $A_{\max}$, who has access to all physical transformations. Then, every transformation is in the equivalence class of the identity transformation, meaning that system $S_{A_{\min}}$ has a single transformation. Now, let $\psi_{0}$ be the cyclic state. By the Initialization Requirement, the set ${\mathrm{Deg}}_{A_{\max}}\left(\psi_{0}\right)$ is the whole state space St(S). Hence, every state is equivalent to the state $\psi_{0}$. In other words, $\mathrm{St}\left(S_{A_{\min}}\right)$ contains only one state. ☐

The Initialization Requirement guarantees the validity of causality, thanks to Proposition 10. In addition, the Initialization Requirement is important independently of the causality property. For example, we will use it to formulate an abstract notion of *closed system*.

## 7. The Conservation of Information

In this section, we consider systems where all transformations are *invertible*. In such systems, every transformation can be thought as the result of some deterministic dynamical law. The different transformations in Transf(S) can be interpreted as different dynamics, associated with different values of physical parameters, such as coupling constants or external control parameters.

### 7.1. Logically Invertible vs. Physically Invertible

**Definition** **10.** *A transformation T∈Transf(S) is* logically invertible *iff the map*

(63) $$\begin{array}{c} \hat{T}:\;\mathrm{St}\left(S\right)\to \mathrm{St}\left(S\right)\;,\;\psi \mapsto T\psi \end{array}$$

*is injective.*

Logically invertible transformations can be interpreted as evolutions of the system that preserve the distictness of states. At the fundamental level, one may require that all physical evolutions be logically invertible, a requirement that is sometimes called the *Conservation of Information* [58]. In the following, we will explore the consequences of such requirement:

**Definition** **11** (Logical Conservation of Information)**.** *System S satisfies the Logical Conservation of Information if all transformations in Transf(S) are logically invertible.*

The requirement is well-posed because the invertible transformations form a monoid. Indeed, the identity transformation is logically invertible, and that the composition of two logically invertible transformations is logically invertible.

A special case of logical invertibility is physical invertibility, defined as follows:

**Definition** **12.** *A transformation T∈Transf(S) is* physically invertible *iff there exists another transformation $T^{\prime }\in \mathrm{Transf}\left(S\right)$ such that $T^{\prime }\circ T=I_{S}$.*

Physical invertibility is more than injectivity: not only should the map T be injective on the state space, but also its inverse should be a physical transformation. In light of this observation, we state a stronger version of the Conservation of Information, requiring physical invertibility:

**Definition** **13** (Physical Conservation of Information)**.** *System S satisfies the Physical Conservation of Information if all transformations in Transf(S) are physically invertible.*

The difference between Logical and Physical Conservation of Information is highlighted by the following example:

**Example** **3** (Conservation of Information in closed-system quantum theory)**.** *Let S be a closed quantum system described by a separable, infinite-dimensional Hilbert space $H_{S}$, and let St(S) be the set of pure states, represented as rank-one density matrices*

(64) $$\begin{array}{c} \mathrm{St}\left(S\right)=\left\{|\psi \rangle \langle \psi |\;:\;|\psi \rangle \in H_{S}\;,\;\langle \psi |\psi \rangle =1\right\}\;. \end{array}$$

*One possible choice of transformations is the monoid of isometric channels*

(65) $$\begin{array}{c} \mathrm{Transf}\left(S\right)=\left\{V\cdot V^{†}\;:\;V\in \mathrm{Lin}\left(S\right)\;,\;V^{†}V=I\right\}\;. \end{array}$$

*This choice of transformations satisfies the Logical Conservation of Information, but violates the Physical Conservation of Information because in general the map $V^{†}\cdot V$ fails to be trace-preserving, and therefore fails to be an isometric channel. For example, consider the shift operator*

(66) $$\begin{array}{c} V=\sum_{n=0}^{\infty }|n+1\rangle \langle n|\;. \end{array}$$

*The operator V is an isometry but its left-inverse $V^{†}$ is not an isometry. As a result, the channel $V^{†}\cdot V$ is not an allowed physical transformation according to Equation (65).*

*An alternative choice of physical transformations is the set of unitary channels*

(67) $$\begin{array}{c} \mathrm{Transf}\left(S\right)=\left\{V\cdot V^{†}\;:\;V\in \mathrm{Lin}\left(S\right)\;,\;V^{†}V=VV^{†}=I\right\}\;. \end{array}$$

*With this choice, the Physical Conservation of Information is satisfied: every physical transformation is invertible and the inverse is a physical transformation.*

### 7.2. Systems Satisfying the Physical Conservation of Information

In a system satisfying the Physical Conservation of Information, the transformations are not only physically invertible, but also physically *reversible*, in the following sense:

**Definition** **14.** *A transformation T∈Transf(S) is* physically reversible *iff there exists another transformation $T^{\prime }\in \mathrm{Transf}\left(S\right)$ such that $T^{\prime }\circ T=T\circ T^{\prime }=I_{S}$.*

With the above definition, we have the following:

**Proposition** **12.** *If system S satisfies the Physical Conservation of Information, then every physical transformation is physically reversible. The monoid Transf(S) is a group, hereafer denoted as G(S).*

**Proof.**  Since T is physically invertible, there exists a transformation $T^{\prime }$ such that $T^{\prime }\circ T=I_{S}$. Since the Physical Conservation of Information holds, $T^{\prime }$ must be physically invertible, meaning that there exists a transformation $T^{\prime \prime }$ such that $T^{\prime \prime }\circ T^{\prime }=I_{S}$. Hence, we have

(68) $$\begin{array}{c} T^{\prime \prime }=T^{\prime \prime }\circ \left(T^{\prime }\circ T\right)=\left(T^{\prime \prime }\circ T^{\prime }\right)\circ T=T\;. \end{array}$$

Since $T^{\prime \prime }=T$, the invertibility condition $T^{\prime \prime }\circ T^{\prime }=I_{S}$ becomes $T\circ T^{\prime }=I_{S}$. Hence, T is reversible and Transf(S) is a group. ☐

### 7.3. Subsystems of Systems Satisfying the Physical Conservation of Information

Imagine that an agent *A* acts on a system *S* satisfying the Physical Conservation of Information. We assume that the actions of agent *A* form a subgroup of G(S), denoted as $G_{A}$. The maximal adversary of *A* is the adversary $B=A^{\prime }$, who has access to all transformations in the set

(69) $$\begin{array}{c} G_{B}:=G_{A}^{\prime }=\left\{U_{B}\in G\left(S\right)\;:\;U_{B}\circ U_{A}=U_{A}\circ U_{B}\;,\;\forall U_{A}\in G\left(A\right)\right\}\;. \end{array}$$

It is immediate to see that the set $G_{B}$ is a group. We call it the *adversarial group*.

The equivalence relations used to define subsystems can be greatly simplified. Indeed, it is easy to see that two states $\psi ,\psi^{\prime }\in \mathrm{St}\left(S\right)$ are equivalent for *A* if and only if there exists a transformation $U_{B}\in G_{B}$ such that

(70) $$\begin{array}{c} \psi^{\prime }=U_{B}\psi \;. \end{array}$$

Hence, the states of the subsystem $S_{A}$ are orbits of the group $G_{B}$: for every ψ∈St(S), we have

(71) $$\begin{array}{c} {\mathrm{Tr}}_{B}\left[\psi \right]:=\left\{U_{B}\psi \;:\;U_{B}\in G_{B}\right\}\;. \end{array}$$

Similarly, the degradation of a transformation U∈G(S) yields the orbit

(72) $$\begin{array}{c} {\mathrm{Deg}}_{B}\left(U\right)=\left\{U_{B,1}\circ U\circ U_{B,2}\;:\;U_{B,1},U_{B,2}\in G_{B}\right\}\;. \end{array}$$

It is easy to show that the transformations of the subsystem $S_{A}$ are the orbits of the group $G_{B}$:

(73) $$\begin{array}{c} \mathrm{Transf}\left(S_{A}\right)=\left\{\pi_{A}\left(U\right)\;:\;U\in G_{A}^{\prime \prime }\right\}\;,\;\pi_{A}\left(U\right):=\left\{U_{B}\circ U\;:\;U_{B}\in G_{B}\right\}\;. \end{array}$$

## 8. Closed Systems

Here, we define an abstract notion of “closed systems”, which captures the essential features of what is traditionally called a closed system in quantum theory. Intuitively, the idea is that all the states of the closed system are “pure” and all the evolutions are reversible.

An obvious problem in defining closed system is that our framework does not include a notion of “pure state”. To circumvent the problem, we define the closed systems in the following way:

**Definition** **15.** *System S is* closed *iff it satisfies the Logical Conservation of Information and the Initialiation Requirement, that is, iff*

*1.*  every transformation is logically invertible,
*2.*  there exists a state $\psi_{0}\in \mathrm{St}\left(S\right)$ such that, for every other state ψ∈St(S), one has $\psi =V\psi_{0}$ for some suitable transformation V∈Transf(S).

For a closed system, we nominally say that all the states in St(S) are “pure”, or, more precisely, “dynamically pure”. This definition is generally different from the usual definition of pure states as extreme points of convex sets, or from the compositional definition of pure states as states with only product extensions [77]. First of all, dynamically pure states are *not a subset* of the state space: provided that the right conditions are met, they are *all* the states. Other differences between the usual notion of pure states and the notion of dynamically pure states are highlighted by the following example:

**Example** **4.** *Let S be a system in which all states are of the form $U\rho_{0}U^{†}$, where U is a generic 2-by-2 unitary matrix, and $\rho_{0}\in M_{2}\left(C\right)$ is a fixed 2-by-2 density matrix. For the transformations, we allow all unitary channels $U\cdot U^{†}$. By construction, system S satisfies the initialization Requirement, as one can generate every state from the initial state $\rho_{0}$. Moreover, all the transformations of system S are unitary and therefore the Conservation of Information is satisfied, both at the physical and the logical level. Therefore, the states of system S are dynamically pure. Of course, the states $U\rho_{0}U^{†}$ need not be extreme points of the convex set of all density matrices, i.e., they need not be rank-one projectors. They are so only when the cyclic state $\rho_{0}$ is rank-one.*

*On the other hand, consider a similar example, where*

- *system S is a qubit,*
- *the states are pure states, of the form |ψ〉〈ψ| for a generic unit vector $|\psi \rangle \in C^{2},$*
- *the transformations are unitary channels $V\cdot V^{†}$, where the unitary matrix V has* real *entries.*

*Using the Bloch sphere picture, the physical transformations are rotations around the y axis. Clearly, the Initialization Requirement is not satisfied because there is no way to generate arbitrary points on the sphere using only rotations around the y-axis. In this case, the states of S are pure in the convex set sense, but not dynamically pure.*

For closed systems satisfying the Physical Conservation of Information, every pair of pure states are interconvertible:

**Proposition** **13** (Transitive action on the pure states)**.** *If system S is closed and satisfies the Physical Conservation of Information, then, for every pair of states $\psi ,\psi^{\prime }\in \mathrm{St}\left(S\right),$ there exists a reversible transformation U∈G(S) such that $\psi^{\prime }=U\psi$.*

**Proof.**  By the Initialization Requirement, one has $\psi =V\psi_{0}$ and $\psi^{\prime }=V^{\prime }\psi_{0}$ for suitable $V,V^{\prime }\in \mathrm{Transf}\left(S\right)$. By the Physical Conservation of Information, all the tranformations in Transf(S) are physically reversible. Hence, $\psi^{\prime }=V^{\prime }\circ V^{-1}\psi =U\psi$, having defined $U=V^{\prime }\circ V^{-1}$. ☐

The requirement that all pure states be connected by reversible transformations has featured in many axiomatizations of quantum theory, either directly [5,44,45,46], or indirectly as a special case of other axioms [42,48]. Comparing our framework with the framework of general probabilistic theories, we can see that the dynamical definition of pure states refers to a rather specific situation, in which all pure states are connected, either to each other (in the case of physical reversibility) or with to a fixed cyclic state (in the case of logical reversibility).

## 9. Purification

Here, we show that closed systems satisfying the Physical Conservation of Information also satisfy the purification property [8,12,13,15,49,50,51], namely the property that every mixed state can be modelled as a pure state of a larger system in a canonical way. Under a certain regularity assumption, the same holds for closed systems satisfying only the Logical Conservation of Information.

### 9.1. Purification in Systems Satisfying the Physical Conservation of Information

**Proposition** **14** (Purification)**.** *Let S be a closed system satisfying the Physical Conservation of Information. Let A be an agent in S, and let $B=A^{\prime }$ be its maximal adversary. Then, for every state $\rho \in \mathrm{St}(S_{A})$, there exists a pure state ψ∈St(S), called the* purification of ρ*, such that $\rho ={\mathrm{Tr}}_{B}\left[\psi \right]$. Moreover, the purification of ρ is* essentially unique *: if $\psi^{\prime }\in \mathrm{St}\left(S\right)$ is another pure state with ${\mathrm{Tr}}_{B}\left[\psi \right]=\rho$, then there exists a reversible transformation $U_{B}\in G_{B}$ such that $\psi^{\prime }=U_{B}\psi$.*

**Proof.**  By construction, the states of system $S_{A}$ are orbits of states of system *S* under the adversarial group $G_{B}$. By Equation (71), every two states $\psi ,\psi^{\prime }\in \mathrm{St}\left(S\right)$ in the same orbit are connected by an element of $G_{B}$. ☐

Note that the notion of purification used here is more general than the usual notion of purification in quantum information and quantum foundations. The most important difference is that system $S_{A}$ need not be a factor in a tensor product. Consider the example of the coherent superpositions vs. classical mixtures (Section 4.3). There, systems $S_{A}$ and $S_{B}$ coincide, their states are classical probability distributions, and the purifications are coherent superpositions. Two purifications of the same classical state $p=(p_{1},p_{2},\cdots ,p_{d})$ are two rank-one projectors |ψ〉〈ψ| and $|\psi^{\prime }\rangle \langle \psi^{\prime }|$ corresponding to unit vectors of the form

(74) $$\begin{array}{c} |\psi \rangle =\sum_{n}\;\sqrt{p_{n}}\;e^{i\theta_{n}}\;|n\rangle \;\mathrm{and}\;|\psi^{\prime }\rangle =\sum_{n}\;\sqrt{p_{n}}\;e^{i\theta_{n}^{\prime }}\;|n\rangle \;. \end{array}$$

One purification can be obtained from the other by applying a diagonal unitary matrix. Specifically, one has

(75) $$\begin{array}{c} |\psi^{\prime }\rangle =U_{B}|\psi \rangle \;\mathrm{with}\;\;U_{B}=\sum_{n}e^{i(\theta_{n}^{\prime }-\theta_{n})}\;|n\rangle \langle n|\;. \end{array}$$

For finite dimensional quantum systems, the notion of purification proposed here encompasses both the notion of entanglement and the notion of coherent superposition. The case of infinite dimensional systems will be discussed in the next subsection.

### 9.2. Purification in Systems Satisfying the Logical Conservation of Information

For infinite dimensional quantum systems, every density matrix can be purified, but not all purifications are connected by reversible transformations. Consider for example the unit vectors

(76) $$\begin{array}{c} {|\psi \rangle }_{AB}=\sqrt{1-x^{2}}\;\sum_{n=0}^{\infty }\;x^{n}{\;|n\rangle }_{A}⊗{|n\rangle }_{B}\;\mathrm{and}\;|\psi^{\prime }\rangle_{AB}=\sqrt{1-x^{2}}\;\sum_{n=0}^{\infty }\;x^{n}{\;|n\rangle }_{A}⊗{|n+1\rangle }_{B}\;, \end{array}$$

for some x∈[0,1).

For every fixed x≠0, there is one and only one operator $V_{B}$ satisfying the condition $|\psi^{\prime }\rangle_{AB}=\left(I_{A}⊗V_{B}\right){|\psi \rangle }_{AB}$, namely the shift operator $V_{B}=\sum_{n=0}^{\infty }\left|n+1\rangle \langle n\right|$. However, $V_{B}$ is only an isometry, but not a unitary. This means that, if we define the states of system $S_{A}$ as equivalence classes of pure states under local unitary equivalence, the two states |ψ〉〈ψ| and $|\psi^{\prime }\rangle \langle \psi^{\prime }|$ would end up into two different equivalence classes.

One way to address the problem is to relax the requirement of reversibility and to consider the monoid of isometries, defining

(77) $$\begin{array}{c} \mathrm{Transf}\left(S\right):=\{V\cdot V^{†}\;:\;V\in \mathrm{Lin}\left(S\right)\;,\;V^{†}V=I\}\;. \end{array}$$

Given two purifications of the same state, say |ψ〉 and $|\psi^{\prime }\rangle$, it is possible to show that at least one of the following possibilities holds:

1. $|\psi^{\prime }\rangle =\left(I_{A}⊗V_{B}\right)\;|\psi \rangle$ for some isometry $V_{B}$ acting on system $S_{B},$
2. $|\psi \rangle =\left(I_{A}⊗V_{B}\right)\;|\psi^{\prime }\rangle$ for some isometry $V_{B}$ acting on system $S_{B}$.

Unfortunately, this uniqueness property is not automatically valid in every system satisfying the Logical Conservation of Information. Still, we will now show a regularity condition, under which the uniqueness property is satisfied:

**Definition** **16.** *Let S be a system satisfying the Logical Conservation of Information, let M⊆Transf(S) be a monoid, and let ${\mathrm{Deg}}_{M}\left(\psi \right)$ be the set defined by*

(78) $$\begin{array}{c} {\mathrm{Deg}}_{M}\left(\psi \right)=\left\{V\;\psi \;:\;V\in M\right\}\;. \end{array}$$

*We say that the monoid M⊆Transf(S) is* regular *iff*

*1.*  for every pair of states $\psi ,\psi^{\prime }\in \mathrm{St}\left(S\right)$, the condition ${\mathrm{Deg}}_{M}\left(\psi \right)\cap {\mathrm{Deg}}_{M}\left(\psi^{\prime }\right)\neq \emptyset$ implies that there exists a transformation U∈M such that $\psi^{\prime }=U\psi$ or $\psi =U\psi^{\prime }$,
*2.*  for every pair of transformations $V,V^{\prime }\in M$, there exists a transformation W∈M such that $V=W\circ V^{\prime }$ or $V^{\prime }=W\circ V$.

The regularity conditions are satisfied in quantum theory by the monoid of isometries.

**Example** **5** (Isometric channels in quantum theory)**.** *Let S be a quantum system with separable Hilbert space H, of dimension d≤∞. Let St(S) the set of all pure quantum states, and let Transf(S) be the monoid of all isometric channels.*

*We now show that the monoid M=Transf(S) is regular. The first regularity condition is immediate because for every pair of unit vectors |ψ〉 and $|\psi^{\prime }\rangle$ there exists an isometry (in fact, a unitary) V such that $|\psi^{\prime }\rangle =U|\psi \rangle$. Trivially, this implies the relation $|\psi^{\prime }\rangle \langle \psi^{\prime }\left|=U|\psi \rangle \langle \psi \right|U^{†}$ at the level of quantum states and isometric channels.*

*Let us see that the second regularity condition holds. Let $V,V^{\prime }\in \mathrm{Lin}\left(H\right)$ be two isometries on H, and let ${\{|i\rangle \}}_{i=1}^{d}$ be the standard basis for H. Then, the isometries V and $V^{\prime }$ can be written as*

(79) $$\begin{array}{c} V=\sum_{i=1}^{d}|\phi_{i}\rangle \langle i|\;\mathrm{and}\;V^{\prime }=\sum_{i}|\phi_{i}^{\prime }\rangle \langle i|\;, \end{array}$$

*where $\{|\phi_{i}\rangle {\}}_{i=1}^{d}$ and $\{|\phi_{i}^{\prime }\rangle {\}}_{i=1}^{d}$ are orthonormal vectors (not necessarily forming bases for the whole Hilbert space H). Define the subspaces $S=\mathrm{Span}\{|\phi_{i}\rangle {\}}_{i=1}^{d}$ and $S^{\prime }=\mathrm{Span}\{|\phi_{i}^{\prime }\rangle {\}}_{i=1}^{d}$, and let $\{|\psi_{j}\rangle {\}}_{j=1}^{r}$ and $\{|\psi_{j}^{\prime }\rangle {\}}_{j=1}^{r^{\prime }}$ be orthonormal bases for the orthogonal complements $S^{⊥}$ and $S^{\prime ⊥}$, respectively. If $r\leq r^{\prime }$, we define the isometry*

(80) $$\begin{array}{c} W=\left(\sum_{i=1}^{d}|\phi_{i}^{\prime }\rangle \langle \phi_{i}|\right)+\left(\sum_{j=1}^{r}|\psi_{j}^{\prime }\rangle \langle \psi_{j}|\right)\;, \end{array}$$

*and we obtain the condition $V^{\prime }=WV$. Alternatively, if $r^{\prime }\leq r$, we can define the isometry*

(81) $$\begin{array}{c} W=\left(\sum_{i=1}^{d}|\phi_{i}\rangle \langle \phi_{i}^{\prime }|\right)+\left(\sum_{j=1}^{r}|\psi_{j}\rangle \langle \psi_{j}^{\prime }|\right)\;, \end{array}$$

*and we obtain the condition $V=WV^{\prime }$. At the level of isometric channels, we obtained the condition $V^{\prime }=W\circ V$ or the condition $V=W\circ V^{\prime }$, with $V\left(\cdot \right)=V\cdot V^{†}$, $V^{\prime }\left(\cdot \right)=V^{\prime }\cdot V^{\prime †}$, and $W\left(\cdot \right)=W\cdot W^{†}$.*

*The fact that the monoid of all isometric channels is regular implies that other monoids of isometric channels are also regular. For example, if the Hilbert space H has the tensor product structure $H=H_{A}⊗H_{B}$, then the monoid of local isometric channels, defined by isometries of the form $I_{A}⊗V_{B}$, is regular. More generally, if the Hilbert space is decomposed as*

(82) $$\begin{array}{c} H=\underset{k}{⨁}\;\left(H_{A,k}⊗H_{B,k}\right)\;, \end{array}$$

*then the monoid of isometric channels generated by isometries of the form*

(83) $$\begin{array}{c} V=\underset{k}{⨁}\;\left(I_{A,k}⊗V_{B,k}\right) \end{array}$$

*is regular.*

We are now in position to derive the purification property for general closed systems:

**Proposition** **15.** *Let S be a closed system. Let A be an agent and let $B=A^{\prime }$ be its maximal adversary. If Act(B;S) is a regular monoid, the condition ${\mathrm{Tr}}_{B}\left[\psi \right]={\mathrm{Tr}}_{B}\left[\psi^{\prime }\right]$ implies that there exists some invertible transformation $V_{B}\in \mathrm{Transf}\left(B;S\right)$ such that the relation $\psi^{\prime }=V_{B}\psi$ or the relation $\psi =V_{B}\psi^{\prime }$ holds.*

The proof is provided in Appendix H. In conclusion, we obtained the following

**Corollary** **1** (Purification)**.** *Let S be a closed system, let A be an agent in S, and let $B=A^{\prime }$ be its maximal adversary. If the monoid Act(B;S) is regular, then every state $\rho \in \mathrm{St}(S_{A})$ has a purification ψ∈St(S), i.e., a state such that $\rho ={\mathrm{Tr}}_{B}\left[\psi \right]$. Moreover, the purification is essentially unique: if $\psi^{\prime }\in \mathrm{St}\left(S\right)$ is another state with ${\mathrm{Tr}}_{B}\left[\psi \right]=\rho$, then there exists a reversible transformation $V_{B}\in \mathrm{Act}\left(B;S\right)$ such that the relation $\psi^{\prime }=V_{B}\psi$ or the relation $\psi =V_{B}\psi^{\prime }$ holds.*

## 10. Example: Group Representations on Quantum State Spaces

We conclude the paper with a macro-example, involving group representations in closed-system quantum theory. The point of this example is to illustrate the general notion of purification introduced in this paper and to characterize the sets of mixed states associated with different agents.

As system *S*, we consider a quantum system with Hilbert space $H_{S}$, possibly of infinite dimension. We let St(S) be the set of pure quantum states, and let G(S) be the group of all unitary channels. With this choice, the total system is closed and satisfies the Physical Conservation of Information.

Suppose that agent *A* is able to perform a group of transformations, such as e.g., the group of phase shifts on a harmonic oscillator, or the group of rotations of a spin *j* particle. Mathematically, we focus our attention on unitary channels arising from some representation of a given compact group G. Denoting the representation as $U:G\to \mathrm{Lin}\left(H_{S}\right)\;,g\mapsto U_{g}$, the group of Alice’s actions is

(84) $$\begin{array}{c} G_{A}=\left\{U_{g}\left(\cdot \right)=U_{g}\cdot U_{g}^{†}:\;g\in G\right\}\;. \end{array}$$

The maximal adversary of *A* is the agent $B=A^{\prime }$ who is able to perform all unitary channels V that commute with those in $G_{A}$, namely, the unitary channels in the group

(85) $$\begin{array}{c} G_{B}:=\left\{V\in G\left(S\right)\;:\;V\circ U_{g}=U_{g}\circ V\;\forall g\in G\right\}\;. \end{array}$$

Specifically, the channels V correspond to unitary operators *V* satisfying the relation

(86) $$\begin{array}{c} VU_{g}=\omega \left(V,g\right)\;U_{g}V\;\forall g\in G\;, \end{array}$$

where, for every fixed *V*, the function ω(V,·):G→C is a multiplicative character, i.e., a one-dimensional representation of the group G.

Note that, if two unitaries *V* and *W* satisfy Equation (86) with multiplicative characters ω(V,·) and ω(W,·), respectively, then their product VW satisfies Equation (86) with multiplicative character ω(VW,·)=ω(V,·)ω(W,·). This means that the function $\omega :G_{B}\times G\to C$ is a multiplicative *bicharacter:* ω(V,·) is a multiplicative character for G for every fixed $V\in G_{B}$, and, at the same time, ω(·,g) is a multiplicative character for $G_{B}$ for every fixed g∈G.

The adversarial group $G_{B}$ contains the commutant of the representation $U:g\mapsto U_{g}$, consisting of all the unitaries *V* such that

(87) $$\begin{array}{c} VU_{g}=U_{g}V\;\forall g\in G\;. \end{array}$$

The unitaries in the commutant satisfy Equation (86) with the trivial multiplicative character ω(V,g)=1, ∀g∈G. In general, the adversarial group may contain other unitary operators, corresponding to non-trivial multiplicative characters. The full characterization of the adversarial group is provided by the following theorem:

**Theorem** **3.** *Let G be a compact group, let U:G→Lin(H) be a projective representation of G, and let $G_{A}$ be the group of channels $G_{A}:=\left\{U_{g}\cdot U_{g}^{†}\;\;g\in G\right\}$. Then, the adversarial group $G_{B}$ is isomorphic to the semidirect product $A⋉U^{\prime }$, where $U^{\prime }$ is the commutant of the set $\left\{U_{g}\;:g\in G\right\}$, and A is an Abelian subgroup of the group of permutations of Irr(U), the set of irreducible representations contained in the decomposition of the representation $U_{g}$.*

The proof is provided in Appendix I, and a simple example is presented in Appendix J.

In the following, we will illustrate the construction of the state space $S_{A}$ in a the prototypical example where the group G is a compact connected Lie group.

### Compact Connected Lie Groups

When G is a compact connected Lie group, the characterization of the adversarial group is simplified by the following theorem:

**Theorem** **4.** *If G is a compact connected Lie group, then the Abelian subgroup A of Theorem 3 is trivial, and all the solutions of Equation (86) have ω(V,g)=1∀g∈G.*

The proof is provided in Appendix K.

For compact connected Lie groups, the the adversarial group coincides exactly with the commutant of the representation $U:G\to \mathrm{Lin}(H_{S})$. An explicit expression can be obtained in terms of the isotypic decomposition [78]

(88) $$\begin{array}{c} U_{g}=\underset{j\in \mathrm{Irr}(U)}{⨁}\;\left(U_{g}^{\left(j\right)}⊗I_{M_{j}}\right)\;, \end{array}$$

where Irr(U) is the set of irreducible representations (irreps) of G contained in the decomposition of *U*, $U^{\left(j\right)}:g\mapsto U_{g}^{\left(j\right)}$ is the irreducible representation of G acting on the representation space $R_{j}$, and $I_{M_{j}}$ is the identity acting on the multiplicity space $M_{j}$. From this expression, it is clear that the adversarial group $G_{B}$ consists of unitary gates *V* of the form

(89) $$\begin{array}{c} V=\underset{j\in \mathrm{Irr}(U)}{⨁}\left(I_{R_{j}}⊗V_{j}\right)\;, \end{array}$$

where $I_{R_{j}}$ is the identity operator on the representation space $R_{j}$, and $V_{j}$ is a generic unitary operator on the multiplicity space $M_{j}$.

In general, the agents *A* and $B=A^{\prime }$ do not form a dual pair. Indeed, it is not hard to see that the maximal adversary of *B* is the agent $C=A^{\prime \prime }$ that can perform every unitary channel $U\left(\cdot \right)=U\cdot U^{†},$ where *U* is a unitary operator of the form

(90) $$\begin{array}{c} U=\underset{j\in \mathrm{Irr}(U)}{⨁}\;\left(U_{j}⊗I_{M_{j}}\right)\;, \end{array}$$

$U_{j}$ being a generic unitary operator on the representation space $R_{j}$. When *A* and *B* form a dual par, the groups $G_{A}$ and $G_{B}$ are sometimes called *gauge groups* [79].

It is now easy to characterize the subsystem $S_{A}$. Its states are equivalence classes of pure states under the relation $\left|\psi \rangle \langle \psi \right|{≃}_{A}|\psi^{\prime }\rangle \langle \psi^{\prime }|$ iff

(91) $$\begin{array}{c} \exists U_{B}\in G_{B}\;\mathrm{such}\;\mathrm{that}\;|\psi^{\prime }\rangle =U_{B}|\psi \rangle \;. \end{array}$$

It is easy to see that two states in the same equivalence class must satisfy the condition

(92) $$\begin{array}{c} {\mathrm{Tr}}_{B}(|\psi^{\prime }\rangle \langle \psi^{\prime }|)={\mathrm{Tr}}_{B}(|\psi \rangle \langle \psi |)\;, \end{array}$$

where the “partial trace over agent *B*” is ${\mathrm{Tr}}_{B}$ is the map

(93) $$\begin{array}{c} {\mathrm{Tr}}_{B}\left(\rho \right):=\underset{j\in \mathrm{Irr}(U)}{⨁}\;{\mathrm{Tr}}_{M_{j}}\left[\Pi_{j}\;\rho \;\Pi_{j}\right]\;, \end{array}$$

$\Pi_{j}$ being the projector on the subspace $R_{j}⊗M_{j}$.

Conversely, it is possible to show that the state ${\mathrm{Tr}}_{B}(|\psi \rangle \langle \psi |)$ completely identifies the equivalence class $[|\psi \rangle {\langle \psi |]}_{A}$.

**Proposition** **16.** *Let $\left|\psi \rangle ,\right|\psi^{\prime }\rangle \in H_{S}$ be two unit vectors such that ${\mathrm{Tr}}_{B}(|\psi \rangle \langle \psi |)={\mathrm{Tr}}_{B}(|\psi^{\prime }\rangle \langle \psi^{\prime }|)$. Then, there exists a unitary operator $U_{B}\in G_{B}$ such that $|\psi^{\prime }\rangle =U_{B}|\psi \rangle$.*

The proof is provided in Appendix L.

We have seen that the states of system $S_{A}$ are in one-to-one correspondence with the density matrices of the form ${\mathrm{Tr}}_{B}(|\psi \rangle \langle \psi |)$, where $|\psi \rangle \in H_{S}$ is a generic pure state. Note that the rank of the density matrices $\rho_{j}$ in Equation (A109) cannot be larger than the dimensions of the spaces $R_{j}$ and $M_{j}$, denoted as $d_{R_{j}}$ and $d_{M_{j}}$, respectively. Taking this fact into account, we can represent the states of $S_{A}$ as

(94) $$\begin{array}{c} \mathrm{St}\left(S_{A}\right)≃\left\{\rho =\underset{j\in \mathrm{Irr}(U)}{⨁}\;p_{j}\;\rho_{j}\;:\;\rho_{j}\in \mathrm{QSt}\left(R_{j}\right)\;,\;\mathrm{Rank}\left(\rho_{j}\right)\leq \min\left\{d_{R_{j}},d_{M_{j}}\right\}\right\}\;, \end{array}$$

where $\left\{p_{j}\right\}$ is a generic probability distribution. The state space of system $S_{A}$ is *not convex*, unless the condition

(95) $$\begin{array}{c} d_{M_{j}}\geq d_{R_{j}}\;\forall j\in \mathrm{Irr}\left(U\right)\; \end{array}$$

is satisfied. Basically, in order to obtain a convex set of density matrices, we need the total system *S* to be “sufficiently large” compared to its subsystem $S_{A}$. This observation is a clue suggesting that the standard convex framework could be considered as the effective description of subsystems of “large” closed systems.

Finally, note that, in agreement with the general construction, the pure states of system *S* are *“purifications"* of the states of the system $S_{A}$. Every state of system $S_{A}$ can be obtained from a pure state of system *S* by *“tracing out"* system $S_{B}$. Moreover, every two purifications of the same state are connected by a unitary transformation in $G_{B}$.

## 11. Conclusions

In this paper, we adopted rather minimalistic framework, in which a single physical system was described solely in terms of states and transformations, without introducing measurements. Or at least, without introducing measurements *in an explicit way*: of course, one could always interpret certain transformations as “measurement processes", but this interpretation is not necessary for any of the conclusions drawn in this paper.

Our framework can be interpreted in two ways. One way is to think of it as a fragment of the larger framework of operational-probabilistic theories [8,11,12,13], in which systems can be freely composed and measurements are explicitly described. The other way is to regard our framework as a dynamicist framework, meant to describe physical systems *per se*, independently of any observer. Both approaches are potentially fruitful.

On the operational-probabilistic side, it is interesting to see how the definition of subsystem adopted in this paper interacts with probabilities. For example, we have seen in a few examples that the state space of a subsystem is not always convex: convex combination of allowed states are not necessarily allowed states. It is then natural to ask: under which condition is convexity retrieved? In a different context, the non-trivial relation between convexity and the dynamical notion of system has been emerged in a work of Galley and Masanes [80]. There, the authors studied alternatives to quantum theory where the closed systems have the same states and the same dynamics of closed quantum systems, while the measurements are different from the quantum measurements. Among these theories, they found that quantum theory is the only theory where subsystems have a convex state space. These and similar clues are an indication that the interplay between dynamical notions and probabilistic notions plays an important role in determining the structure of physical theories. Studying this interplay is a promising avenue of future research.

On the opposite end of the spectrum, it is interesting to explore how far the measurement-free approach can reach. An interesting research project is to analyze the notions of subsystem, pure state, and purification, in the context of algebraic quantum field theory [22] and quantum statistical mechanics [32]. This is important because the notion of pure state as an extreme point of the convex set breaks down for type III von Neumann algebras [81], whereas the notions used in this paper (commutativity of operations, cyclicity of states) would still hold. Another promising clue is the existence of dual pairs of non-overlapping agents, which amounts to the requirement that the set of operations of each agent has trivial center and coincides with its double commutant. A similar condition plays an important role in the algebraic framework, where the operator algebras with trivial center are known as factors, and are at the basis of the theory of von Neumann algebras [82,83].

Finally, another interesting direction is to enrich the structure of system with additional features, such as a metric, quantifying the proximity of states. In particular, one may consider a strengthened formulation of the Conservation of Information, in which the physical transformations are required not only to be invertible, but also to preserve the distances. It is then interesting to consider how the metric on the pure states of the whole system induces a metric on the subsystems, and to search for relations between global metric and local metric. Also in this case, there is a promising precedent, namely the work of Uhlmann [84], which led to the notion of fidelity [85]. All these potential avenues of future research suggest that the notions investigated in this work may find application in a variety of different contexts, and for a variety of interpretational standpoints.

## Appendix A. Proof That Definitions (20) and (21) Are Well-Posed

We give only the proof for definition (20), as the other proof follows the same argument.

**Proposition** **A1.**
*If the transformations $S,\tilde{S},T,\tilde{T}\in \mathrm{Act}{\left(A;S\right)}^{\prime \prime }$ are such that ${\left[S\right]}_{A}={\left[\tilde{S}\right]}_{A}$ and ${\left[T\right]}_{A}={\left[\tilde{T}\right]}_{A}$, then ${\left[S\circ T\right]}_{A}={\left[\tilde{S}\circ \tilde{T}\right]}_{A}$.*

**Proof.** Let $\left(S_{1},S_{2},\cdots ,S_{m}\right)\subset \mathrm{Act}{\left(A;S\right)}^{\prime \prime }$ and $\left(T_{1},T_{2},\cdots ,T_{n}\right)\subset \mathrm{Act}{\left(A;S\right)}^{\prime \prime }$ be two finite sequences such that

(A1) $$\begin{array}{c} S_{1}=S\;,\;S_{m}=\tilde{S}\;,\;{\mathrm{Deg}}_{A^{\prime }}\left(S_{i}\right)\cap {\mathrm{Deg}}_{A^{\prime }}\left(S_{i+1}\right)\neq \emptyset \;\;\forall i\in \left\{1,\cdots ,m-1\right\},\\ T_{1}=T\;,\;T_{n}=\tilde{T}\;,\;{\mathrm{Deg}}_{A^{\prime }}\left(T_{j}\right)\cap {\mathrm{Deg}}_{A^{\prime }}\left(T_{j+1}\right)\neq \emptyset \;\;\forall j\in \left\{1,\cdots ,n-1\right\}. \end{array}$$

Without loss of generality, we assume that the two finite sequences have the same length m=n. When this is not the case, one can always add dummy entries and ensure that the two sequences have the same length: for example, if m<n, one can always define $S_{i}:=S_{m}$ for all i∈{m+1,⋯,n}. Equation (A1) mean that for every *i* and *j* there exist transformations $B_{i},\tilde{B_{i}},C_{j},\tilde{C_{j}}\in \mathrm{Act}{\left(A;S\right)}^{\prime }$ such that

(A2) $$\begin{array}{cc} B_{i}\circ S_{i} & =\tilde{B_{i}}\circ S_{i+1},\\ C_{j}\circ T_{j} & =\tilde{C_{j}}\circ T_{j+1}. \end{array}$$

Using the above equalities for i=j, and using the fact that transformations in $\mathrm{Act}{\left(A;S\right)}^{\prime }$ commute with transformations in $\mathrm{Act}{\left(A;S\right)}^{\prime \prime }$, we obtain

(A3) $$\begin{array}{cc} \left(B_{i}\circ C_{i}\right)\circ \left(S_{i}\circ T_{i}\right) & =\left(B_{i}\circ S_{i}\right)\circ \left(C_{i}\circ T_{i}\right)\\ & =\left(\tilde{B_{i}}\circ S_{i+1}\right)\circ \left(\tilde{C_{i}}\circ T_{i+1}\right)\\ & =\left(\tilde{B_{i}}\circ \tilde{C_{i}}\right)\circ \left(S_{i+1}\circ T_{i+1}\right)\;. \end{array}$$

In short, we proved that

(A4) $$\begin{array}{c} {\mathrm{Deg}}_{A^{\prime }}\left(S_{i}\circ T_{i}\right)\cap {\mathrm{Deg}}_{A^{\prime }}\left(S_{i+i}\circ T_{i+1}\right)\neq \emptyset \;\;\forall i\in \left\{1,\cdots ,n-1\right\}\;. \end{array}$$

To conclude, observe that the sequence $\left(S_{1}\circ T_{1},S_{2}\circ T_{2},\cdots ,S_{n}\circ T_{n}\right)$ satisfies $S_{1}\circ T_{1}=S\circ T$, $S_{n}\circ T_{n}=\tilde{S}\circ \tilde{T}$, and Equation (A4). By definition, this means that the transformations S∘T and $\tilde{S}\circ \tilde{T}$ are in the same equivalence class. ☐

## Appendix B. The Commutant of the Local Channels

Here, we show that the commutant of the quantum channels of the form $A⊗I_{B}$ consists of quantum channels of the form $I_{A}⊗B$.

Let C∈Chan(S) be a quantum channel that commutes with all channels of the form $A⊗I_{B}$, with A∈Chan(A). For a fixed unit vector $|\alpha \rangle \in H_{A}$, consider the erasure channel $A_{\alpha }\in \mathrm{Chan}\left(A\right)$ defined by

(A5) $$\begin{array}{c} A_{\alpha }\left(\rho \right)=|\alpha \rangle \langle \alpha |\;\mathrm{Tr}\left[\rho \right]\;\forall \rho \in \mathrm{Lin}\left(A\right)\;. \end{array}$$

Then, the commutation condition $C\circ \left(A_{\alpha }⊗I_{B}\right)=\left(A_{\alpha }⊗I_{B}\right)\circ C$ implies

(A6) $$\begin{array}{cc} C\left(|\alpha \rangle \langle \alpha |⊗|\beta \rangle \langle \beta |\right) & =C\left[\left(A_{\alpha }⊗I_{B}\right)\left(|\alpha \rangle \langle \alpha |⊗|\beta \rangle \langle \beta |\right)\right]\\ & =\left(A_{\alpha }⊗I_{B}\right)\left[C\left(|\alpha \rangle \langle \alpha |⊗|\beta \rangle \langle \beta |\right)\right]\\ & =\left|\alpha \rangle \langle \alpha \right|⊗{\mathrm{Tr}}_{A}\left[C\left(|\alpha \rangle \langle \alpha |⊗|\beta \rangle \langle \beta |\right)\right]\;\forall |\beta \rangle \in H_{B}\;. \end{array}$$

Tracing over *B* on both sides of Equation (A6), we obtain

(A7) $$\begin{array}{c} {\mathrm{Tr}}_{B}\left[C\left(|\alpha \rangle \langle \alpha |⊗|\beta \rangle \langle \beta |\right)\right]=|\alpha \rangle \langle \alpha |\;. \end{array}$$

The above relation implies that the state $C\left(|\alpha \rangle \langle \alpha |⊗|\beta \rangle \langle \beta |\right)$ is of the form

(A8) $$\begin{array}{cc} C\left(|\alpha \rangle \langle \alpha |⊗|\beta \rangle \langle \beta |\right) & =|\alpha \rangle \langle \alpha |⊗B(|\beta \rangle \langle \beta |)\;, \end{array}$$

for some suitable channel B∈Chan(B). Since |α〉 and |β〉 are arbitrary, we obtained $C=I_{A}⊗B$.

## Appendix C. Subsystems Associated to Finite Dimensional Von Neumann Algebras

Here, we prove the statements made in the main text about quantum channels with Kraus operators in a given algebra.

### Appendix C.1. The Commutant of *Chan(A)*

The purpose of this subsection is to prove the following theorem:

**Theorem** **A1.**
*Let A be a von Neumann subalgebra of $M_{d}\left(C\right)$, d<∞, and let Chan(A) be the set of quantum channels with Kraus operators in A. Then, the commutant of Chan(A) is the set of channels with Kraus operators in the algebra $A^{\prime }$. In formula,*

(A9) $$\begin{array}{c} \mathrm{Chan}{\left(A\right)}^{\prime }=\mathrm{Chan}\left(A^{\prime }\right)\;. \end{array}$$

The proof consists of a few lemmas, provided in the following.

**Lemma** **A1.**
*Every channel $D\in \mathrm{Chan}{\left(A\right)}^{\prime }$ must satisfy the condition*

(A10) $$\begin{array}{c} P_{l}\circ D\circ P_{k}=0\;\forall l\neq k\;, \end{array}$$

*where $P_{k}$ is the CP map $P_{k}\left(\cdot \right):=\Pi_{k}\cdot \Pi_{k}$, and $\Pi_{k}$ is the projector on the subspace $H_{A_{k}}⊗H_{B_{k}}$ in Equation (31).*

**Proof.** Consider the quantum channel C∈Chan(A) defined as

(A11) $$\begin{array}{c} C:=\underset{k}{⨁}\;\left(|\alpha_{k}\rangle \langle \alpha_{k}|\;{\mathrm{Tr}}_{A_{k}}⊗I_{B_{k}}\right)\circ P_{k}\;, \end{array}$$

where each $|\alpha_{k}\rangle$ is a generic (but otherwise fixed) unit vector in $H_{A_{k}}$ and $I_{B_{k}}$ is the identity map on $\mathrm{Lin}(H_{B_{k}})$. By definition, every channel $D\in \mathrm{Chan}{\left(A\right)}^{\prime }$ must satisfy the condition C∘D=D∘C. In particular, we must have

(A12) $$\begin{array}{cc} D(|\alpha_{k}\rangle \langle \alpha_{k}\left|⊗\right|\beta_{k}\rangle \langle \beta_{k}|) & =\left(D\circ C\right)(|\alpha_{k}\rangle \langle \alpha_{k}\left|⊗\right|\beta_{k}\rangle \langle \beta_{k}|)\\ & =\left(C\circ D\right)(|\alpha_{k}\rangle \langle \alpha_{k}\left|⊗\right|\beta_{k}\rangle \langle \beta_{k}|)\\ & =\underset{l}{⨁}\;\left(|\alpha_{l}\rangle \langle \alpha_{l}|⊗{\mathrm{Tr}}_{A_{l}}\left[\left(P_{l}\circ \;D\right)(|\alpha_{k}\rangle \langle \alpha_{k}\left|⊗\right|\beta_{k}\rangle \langle \beta_{k}|)\;\right]\right). \end{array}$$

Applying the CP map $P_{l}$ on both sides of the above equality, we obtain the relation

(A13) $$\begin{array}{c} \left(P_{l}\circ D\right)(|\alpha_{k}\rangle \langle \alpha_{k}\left|⊗\right|\beta_{k}\rangle \langle \beta_{k}|)=|\alpha_{l}\rangle \langle \alpha_{l}|⊗M_{l}(|\alpha_{k}\rangle \langle \alpha_{k}\left|⊗\right|\beta_{k}\rangle \langle \beta_{k}|)\;, \end{array}$$

where $M_{l}$ is the map from $M_{d}\left(C\right)$ to $\mathrm{Lin}(H_{A_{l}})$ defined as $M_{l}:={\mathrm{Tr}}_{A_{l}}\circ P_{l}\circ D$. Note that the right-hand side of Equation (A13) depends on the choice of vector $|\alpha_{l}\rangle$, which is arbitrary. On the other hand, the left-hand side does not depend on $|\alpha_{l}\rangle$. Hence, the only way that the two sides of Equation (A13) can be equal for k≠l is that they are both equal to 0. Moreover, since $|\alpha_{k}\rangle$ and $|\beta_{k}\rangle$ are arbitrary vectors in $H_{A_{k}}$ and $H_{B_{k}}$, respectively, Equation (A13) implies the relation

(A14) $$\begin{array}{c} \left(P_{l}\circ D\right)\left(\rho \right)=0\;\forall \rho \in \mathrm{Lin}\left(H_{A_{k}}⊗H_{B_{k}}\right)\;,\;\forall l\neq k\;. \end{array}$$

Since ρ is an arbitrary operator in $\mathrm{Lin}(H_{A_{k}}⊗H_{B_{k}})$, we conclude that the relation $P_{l}\circ D\circ P_{k}=0$ holds for every l≠k. ☐

**Lemma** **A2.**
*Every channel $D\in \mathrm{Chan}{\left(A\right)}^{\prime }$ must satisfy the conditions*

(A15) $$\begin{array}{c} D\circ P_{k}=P_{k}\circ D\circ P_{k}\;\forall k\; \end{array}$$

*and*

(A16) $$\begin{array}{c} P_{k}\circ D=P_{k}\circ D\circ P_{k}\;\forall k\;. \end{array}$$

*In short: $D\circ P_{k}=P_{k}\circ D$ for every k.*

**Proof.** Define $D_{k}:=D\circ P_{k}$. Then, the Cauchy–Schwarz inequality yields

(A17) $$\begin{array}{cc} \left|\langle \phi |\Pi_{i}\;D_{k}\left(\rho \right)\;\Pi_{j}\;|\phi \rangle \right| & \leq \sqrt{\langle \phi |\Pi_{i}\;D_{k}\left(\rho \right)\;\Pi_{i}\;|\phi \rangle \;\langle \phi |\Pi_{j}\;D_{k}\left(\rho \right)\;\Pi_{j}\;|\phi \rangle }\\ & \leq \sqrt{\langle \phi |\left(P_{i}\circ D\circ P_{k}\right)\left(\rho \right)\;|\phi \rangle \;\langle \phi |\left(P_{j}\circ D\circ P_{k}\right)\left(\rho \right)\;|\phi \rangle }\;. \end{array}$$

Thanks to Lemma A1, we know the right-hand side is 0 unless i=j=k. Since the vector |ϕ〉 is are arbitrary, the condition $|\langle \phi |\Pi_{i}\;D_{k}\left(\rho \right)\;\Pi_{j}\;|\phi \rangle |=0$ implies the relation $\Pi_{i}\;D_{k}\left(\rho \right)\;\Pi_{j}=0$. Using this fact, we obtain the relation

(A18) $$\begin{array}{cc} \left(D\circ P_{k}\right)\left(\rho \right) & =D_{k}\left(\rho \right)\\ & =\sum_{i,j}\;\Pi_{i}\;D_{k}\left(\rho \right)\;\Pi_{j}\\ & =\;\Pi_{k}\;D_{k}\left(\rho \right)\;\Pi_{k}\\ & =\left(P_{k}\circ D\circ P_{k}\right)\left(\rho \right)\;, \end{array}$$

valid for arbitrary density matrices ρ, and therefore for arbitrary matrices in $M_{d}\left(C\right)$. In conclusion, Equation (A16) holds. The proof of Equation (A15) is analogous to that of Equation (A16), with the only difference that it uses the *adjoint map*, which for a generic linear map $L:\mathrm{Lin}\left(H_{S}\right)\to \mathrm{Lin}\left(H_{S}\right)$ is defined by the relation

(A19) $$\begin{array}{c} \mathrm{Tr}\left[L^{†}\left(O\right)\;\rho \right]:=\mathrm{Tr}\left[O\;L\left(\rho \right)\right]\;\forall O\in M_{d}\left(C\right)\;,\;\forall \rho \in M_{d}\left(C\right)\;. \end{array}$$

Specifically, we define the map ${\tilde{D}}_{k}:=P_{k}\circ D$. Then, we obtain the relation

(A20) $$\begin{array}{cc} \left|\;\langle \phi |\;{\tilde{D}}_{k}\left(\Pi_{i}\rho \Pi_{j}\right)\;|\phi \rangle \right| & =|\mathrm{Tr}\left[{\tilde{D}}_{k}^{†}(|\phi \rangle \langle \phi |)\Pi_{i}\rho \Pi_{j}\right]|\\ & =\left|\mathrm{Tr}\left[\left(\sqrt{{\tilde{D}}_{k}^{†}(|\phi \rangle \langle \phi |)}\Pi_{i}\sqrt{\rho }\right)\;\left(\sqrt{\rho }\Pi_{j}\sqrt{{\tilde{D}}_{k}^{†}(|\phi \rangle \langle \phi |}\right)\right]\right|\\ & \leq \sqrt{\mathrm{Tr}\left[D_{k}^{†}(|\phi \rangle \langle \phi |)\;\Pi_{i}\rho \Pi_{i}\right]\;\mathrm{Tr}\left[D_{k}^{†}(|\phi \rangle \langle \phi |)\;\Pi_{j}\rho \Pi_{j}\right]}\\ & =\sqrt{\langle \phi |\;{\tilde{D}}_{k}\left(\Pi_{i}\rho \Pi_{i}\right)\;|\phi \rangle \;\langle \psi |\;{\tilde{D}}_{k}\left(\Pi_{j}\rho \Pi_{j}\right)\;|\psi \rangle }\\ & =\sqrt{\langle \phi |\;\left(P_{k}\circ D\circ P_{i}\right)\left(\rho \right)\;|\phi \rangle \;\langle \psi |\;\left(P_{k}\circ D\circ P_{j}\right)\left(\rho \right)\;|\psi \rangle }\;, \end{array}$$

where the right-hand side is 0 unless i=j=k (cf. Lemma A2). Since the condition $|\;\langle \phi |\;{\tilde{D}}_{k}\left(\Pi_{i}\rho \Pi_{j}\right)\;|\phi \rangle |=0,\;\forall |\phi \rangle \in H_{S}$ implies the condition ${\tilde{D}}_{k}\left(\Pi_{i}\rho \Pi_{j}\right)=0$, we obtained the relation

(A21) $$\begin{array}{c} {\tilde{D}}_{k}\left(\Pi_{i}\rho \Pi_{j}\right)=0\;\mathrm{unless}\;i=j=k\;. \end{array}$$

Using this fact, we obtain the equality

(A22) $$\begin{array}{cc} \left(P_{k}\circ D\right)\left(\rho \right) & ={\tilde{D}}_{k}\left(\rho \right)\\ & =\sum_{i,j}\;{\tilde{D}}_{k}\left(\Pi_{i}\rho \Pi_{j}\right)\\ & =\left({\tilde{D}}_{k}\circ P_{k}\right)\left(\rho \right)\\ & =\left(P_{k}\circ D\circ P_{k}\right)\left(\rho \right)\;. \end{array}$$

Since the equality holds for every ρ, this proves Equation (A16). ☐

Lemma A2 guarantees that the linear map $D\circ P_{k}$ sends $\mathrm{Lin}(R_{k}⊗M_{k})$ into itself. It is also easy to see that the map $D\circ P_{k}$ has a simple form:

**Lemma** **A3.**
*For every channel $D\in \mathrm{Chan}{\left(A\right)}^{\prime }$, one has*

(A23) $$\begin{array}{c} D\circ P_{k}=\left(I_{A_{k}}⊗B_{k}\right)\circ P_{k}\;\forall k, \end{array}$$

*where $I_{A_{k}}$ is the identity map from $\mathrm{Lin}(H_{A_{k}})$ to itself, and $B_{k}$ is a quantum channel from $\mathrm{Lin}(H_{A_{k}})$ to itself.*

**Proof.** Straightforward extension of the proof in Appendix B. ☐

Using the notion of adjoint, we can now prove the following

**Lemma** **A4.**
*For every channel $D\in \mathrm{Chan}{\left(A\right)}^{\prime }$, the adjoint $D^{†}$ preserves the elements of the algebra A, namely $D^{†}\left(C\right)=C$ for all C∈A.*

**Proof.** Let *C* be a generic element of A. By Equation (31), one has the equality

(A24) $$\begin{array}{c} C=\underset{k}{⨁}\left(C_{k}⊗I_{B_{k}}\right)=\underset{k}{⨁}P_{k}\left(C\right). \end{array}$$

Using Lemma A3 and the definition of adjoint, we obtain

(A25) $$\begin{array}{cc} \mathrm{Tr}[D^{†}\left(C\right)\;\rho ] & =\mathrm{Tr}[C\;D(\rho )]\\ & =\sum_{k}\;\mathrm{Tr}\left[P_{k}\left(C\right)\;D\left(\rho \right)\right]\\ & =\sum_{k}\;\mathrm{Tr}\left[C\;\left(P_{k}\circ D\right)\left(\rho \right)\right]\\ & =\sum_{k}\;\mathrm{Tr}\left[C\;\left(P_{k}\circ D\circ P_{k}\right)\left(\rho \right)\right]\\ & =\sum_{k}\;\mathrm{Tr}\left[P_{k}\left(C\right)\;\left(D\circ P_{k}\right)\left(\rho \right)\;\right]\\ & =\sum_{k}\;\mathrm{Tr}\left\{\left(C_{k}⊗I_{B_{k}}\right)\;\left[\left(I_{A_{k}}⊗B_{k}\right)\circ P_{k}\right]\left(\rho \right)\right\}\;, \end{array}$$

having used Lemma A3 in the last equality. Then, we use the fact that the channel $B_{k}$ is trace-preserving, and therefore its adjoint $B_{k}^{†}$ preserves the identity. Using this fact, we can continue the chain of equalities as

(A26) $$\begin{array}{cc} \mathrm{Tr}[D^{†}\left(C\right)] & =\sum_{k}\;\mathrm{Tr}\left\{\left[C_{k}⊗B_{k}^{†}\left(I_{B_{k}}\right)\right]\;P_{k}\left(\rho \right)\right\}\\ & =\sum_{k}\;\mathrm{Tr}\left[\left(C_{k}⊗I_{B_{k}}\right)\;P_{k}\left(\rho \right)\right]\\ & =\sum_{k}\;\mathrm{Tr}\left[P_{k}\left(C_{k}⊗I_{B_{k}}\right)\;\rho \right]\\ & =\;\mathrm{Tr}\left[\left(\underset{k}{⨁}C_{k}⊗I_{B_{k}}\right)\;\rho \right]\\ & =\mathrm{Tr}[C\rho ]\;, \end{array}$$

having used Equation (A24) in the last equality. Since the equality holds for every density matrix ρ, we proved the equality $D^{†}\left(C\right)=C$. ☐

We are now in position to prove Theorem A1.

**Proof of Theorem A1.** Let D be a quantum channel in $\mathrm{Chan}{\left(A\right)}^{\prime }$. Then, Lemma A4 guarantees that the adjoint $D^{†}$ preserves all operators in the algebra A. Then, a result due to Lindblad [86] guarantees that all the Kraus operators of D belong to the algebra $A^{\prime }$. This proves the inclusion $\mathrm{Chan}{\left(A\right)}^{\prime }\subseteq \mathrm{Chan}\left(A^{\prime }\right)$.

The converse inclusion is immediate: if a channel D belongs to $\mathrm{Chan}(A^{\prime })$, it commutes with all channels in Chan(A) thanks to the block diagonal form of the Kraus operators (cf. Equations (32) and (33)). ☐

### Appendix C.2. States of Subsystems Associated to Finite Dimensional Von Neumann algebras

Here, we provide the proof of Proposition 5, adopting the notation $B:=A^{\prime }$.

The proof uses the following lemma:

**Lemma** **A5** (No signalling condition)**.**
*For every channel D∈Chan(B), one has ${\mathrm{Tr}}_{B}\circ D={\mathrm{Tr}}_{B}$.*

**Proof.** By definition, the partial trace channel ${\mathrm{Tr}}_{B}$ can be written as

(A27) $$\begin{array}{c} {\mathrm{Tr}}_{B}=\underset{k}{⨁}\;\left(I_{A_{k}}⊗{\mathrm{Tr}}_{B_{k}}\right)\circ P_{k}. \end{array}$$

For every channel D∈Chan(B), we have

(A28) $$\begin{array}{cc} {\mathrm{Tr}}_{B}\circ D & =\underset{k}{⨁}\;\left(I_{A_{k}}⊗{\mathrm{Tr}}_{B_{k}}\right)\circ P_{k}\circ D\\ & =\underset{k}{⨁}\;\left(I_{A_{k}}⊗{\mathrm{Tr}}_{B_{k}}\right)\circ \left(I_{A_{k}}⊗B_{k}\right)\circ P_{k}\\ & =\underset{k}{⨁}\;\left[I_{A_{k}}⊗\left({\mathrm{Tr}}_{B_{k}}\circ B_{k}\right)\right]\circ P_{k}\\ & =\underset{k}{⨁}\;\left(I_{A_{k}}⊗{\mathrm{Tr}}_{B_{k}}\right)\circ P_{k}\\ & ={\mathrm{Tr}}_{B}, \end{array}$$

where the second equality follows from Lemma A3, and the third equality follows from the fact that $B_{k}$ is trace-preserving. ☐

**Proof of Proposition 5.** Suppose that ρ and σ are equivalent for *A*. By definition, this means that there exists a finite sequence $\left(\rho_{1},\rho_{2},\cdots ,\rho_{n}\right)$ such that

(A29) $$\begin{array}{c} \rho_{1}=\rho \;,\;\rho_{n}=\sigma \;,\;\mathrm{and}\;{\mathrm{Deg}}_{B}\left(\rho_{i}\right)\cap {\mathrm{Deg}}_{B}\left(\rho_{i+1}\right)\neq \emptyset \;\forall i\in \left\{1,2,\cdots ,n-1\right\}\;. \end{array}$$

The condition of non-trivial intersection implies that, for every i∈{1,2,⋯,n−1}, one has

(A30) $$\begin{array}{c} D_{i}\;\left(\rho_{i}\right)={\tilde{D}}_{i}\;\left(\rho_{i+1}\right)\;, \end{array}$$

where $D_{i}$ and ${\tilde{D}}_{i}$ are two quantum channels in Chan(B). Tracing over B on both sides we obtain the relation

(A31) $$\begin{array}{c} \left({\mathrm{Tr}}_{B}\circ D_{i}\right)\;\left(\rho_{i}\right)=\left({\mathrm{Tr}}_{B}\circ {\tilde{D}}_{i}\right)\;\left(\rho_{i+1}\right)\;, \end{array}$$

and, thanks to Lemma A5, ${\mathrm{Tr}}_{B}\left[\rho_{i}\right]={\mathrm{Tr}}_{B}\left[\rho_{i+1}\right]$. Since the equality holds for every i∈{1,⋯,n−1}, we obtained the condition ${\mathrm{Tr}}_{B}\left[\rho \right]={\mathrm{Tr}}_{B}\left[\sigma \right]$. In summary, if two states ρ and σ are equivalent for *A*, then ${\mathrm{Tr}}_{B}\left[\rho \right]={\mathrm{Tr}}_{B}\left[\sigma \right]$.

To prove the converse, it is enough to define the channel $D_{0}\in \mathrm{Chan}\left(B\right)$ as

(A32) $$\begin{array}{c} D_{0}\left(\rho \right):=\underset{k}{⨁}\;{\mathrm{Tr}}_{B_{k}}\left[P_{k}\left(\rho \right)\right]⊗\beta_{k}\;, \end{array}$$

where each $\beta_{k}$ is a fixed (but otherwise generic) density matrix in $\mathrm{Lin}(H_{B_{k}})$. Now, if the equality ${\mathrm{Tr}}_{B}\left[\rho \right]={\mathrm{Tr}}_{B}\left[\sigma \right]$ holds, then also the equality $D_{0}\left(\rho \right)=D_{0}\left(\sigma \right)$ holds. This proves that the intersection between ${\mathrm{Deg}}_{B}\left(\rho \right)$ and ${\mathrm{Deg}}_{B}\left(\sigma \right)$ is non-empty, and therefore ρ and σ are equivalent for *A*. ☐

### Appendix C.3. Transformations of Subsystems Associated to Finite Dimensional von Neumann algebras

Here, we prove that all transformations of system $S_{A}$ are of the form $A={⨁}_{k}A_{k}$, where each $A_{k}$ is a quantum channel from $\mathrm{Lin}(H_{A_{k}})$ to itself. The proof is based on the following lemmas:

**Lemma** **A6.**
*For every channel C∈Chan(A), one has the relation*

(A33) $$\begin{array}{c} P_{k}\circ C=\left(A_{k}⊗I_{B_{k}}\right)\circ P_{k}\;, \end{array}$$

*where $A_{k}$ is a quantum channel from $\mathrm{Lin}(H_{A_{k}})$ to itself.*

**Proof.** Let

(A34) $$\begin{array}{c} C\left(\rho \right)=\sum_{i}\;C_{i}\;\rho \;C_{i}^{†}\;,\;C_{i}=\underset{k}{⨁}\;\left(C_{ik}⊗I_{B_{k}}\right)\; \end{array}$$

be a Kraus representation of channel C. The preservation of the trace amounts to the condition

(A35) $$\begin{array}{cc} I & =\sum_{i}C_{i}^{†}C_{i}\\ & =\underset{k}{⨁}\left(\sum_{i}C_{ik}^{†}C_{ik}⊗I_{B_{k}}\right)\;, \end{array}$$

which implies

(A36) $$\begin{array}{c} \sum_{i}C_{ik}^{†}C_{I_{k}}=I_{A_{k}}\;\forall k\;. \end{array}$$

Now, we have

(A37) $$\begin{array}{cc} \left(P_{k}\circ C\right)\left(\rho \right) & =\sum_{i}\left(C_{ik}⊗I_{B_{k}}\right)\;P_{k}\left(\rho \right)\;{\left(C_{ik}⊗I_{B_{k}}\right)}^{†}\\ & =\left(A_{k}⊗I_{B_{k}}\right)\;\left[P_{k}\left(\rho \right)\right]\;, \end{array}$$

where the channel $A_{k}$ is defined as

(A38) $$\begin{array}{c} A_{k}\left(\sigma \right):=\sum_{i}C_{ik}\;\sigma \;C_{ik}^{†}\;\forall \sigma \in \mathrm{Lin}\left(H_{A_{k}}\right)\;. \end{array}$$

Since the density matrix ρ in Equation (A37) is arbitrary, we proved the relation $P_{k}\circ C=\left(A_{k}⊗I_{B_{k}}\right)\circ P_{k}$. ☐

**Lemma** **A7.**
*For two channels $C,C^{\prime }\in \mathrm{Chan}\left(A\right)$, let $A_{k}$ and $A_{k}^{\prime }$ be the quantum channels defined in Lemma A6. Then, the following are equivalent:*

1. ${\mathrm{Tr}}_{B}\circ \;C={\mathrm{Tr}}_{B}\circ \;C^{\prime },$
2. *$A_{k}=A_{k}^{\prime }\;$ for every k.*

**Proof.** 2⟹1. For channel C, we have

(A39) $$\begin{array}{cc} {\mathrm{Tr}}_{B}\circ \;C & =\underset{k}{⨁}\;\left(I_{A_{k}}⊗{\mathrm{Tr}}_{B_{k}}\right)\circ P_{k}\circ C\\ & =\underset{k}{⨁}\;\left(I_{A_{k}}⊗{\mathrm{Tr}}_{B_{k}}\right)\circ \left(A_{k}⊗I_{B_{k}}\right)\circ P_{k}\\ & =\underset{k}{⨁}\;\left(A_{k}⊗{\mathrm{Tr}}_{B_{k}}\right)\circ P_{k}\;. \end{array}$$

Similarly, for channel $C^{\prime }$, we have

(A40) $$\begin{array}{c} {\mathrm{Tr}}_{B}\circ \;C^{\prime }=\underset{k}{⨁}\;\left(A_{k}^{\prime }⊗{\mathrm{Tr}}_{B_{k}}\right)\circ P_{k}\;. \end{array}$$

Clearly, if $A_{k}$ and $A_{k}^{\prime }$ are equal for every *k*, then the partial traces ${\mathrm{Tr}}_{B}\circ \;C$ and ${\mathrm{Tr}}_{B}\circ \;C^{\prime }$ are equal. 1⟹2. Suppose that partial traces ${\mathrm{Tr}}_{B}\circ \;C$ and ${\mathrm{Tr}}_{B}\circ \;C^{\prime }$ are equal. Then, Equations (A39) and (A40) imply the equality

(A41) $$\begin{array}{c} \left(A_{k}⊗{\mathrm{Tr}}_{B_{k}}\right)\circ P_{k}=\left(A_{k}^{\prime }⊗{\mathrm{Tr}}_{B_{k}}\right)\circ P_{k}\;\forall k\;. \end{array}$$

In turn, the above equality implies $A_{k}=A_{k}^{\prime }$, ∀k, as one can easily verify by applying both sides of Equation (A41) to a generic product operator $X_{k}⊗Y_{k}$, with $X_{k}\in \mathrm{Lin}\left(H_{A_{k}}\right)$ and $Y_{k}\in \mathrm{Lin}\left(H_{B_{k}}\right)$. ☐

**Lemma** **A8.**
*Two channels $C,C^{\prime }\in \mathrm{Chan}\left(A\right)$ are equivalent for A if and only if ${\mathrm{Tr}}_{B}\circ \;C={\mathrm{Tr}}_{B}\circ \;C^{\prime }$.*

**Proof.** Suppose that C and $C^{\prime }$ are equivalent for *A*. By definition, this means that there exists a finite sequence $\left(C_{1},C_{2},\cdots ,C_{n}\right)\subset \mathrm{Chan}\left(A\right)$ such that

(A42) $$\begin{array}{c} C_{1}=C\;,\;C_{n}=C^{\prime }\;,\;{\mathrm{Deg}}_{B}\left(C_{i}\right)\cap {\mathrm{Deg}}_{B}\left(C_{i+1}\right)\neq \emptyset \;\;\forall i\in \left\{1,\cdots ,n-1\right\}\;. \end{array}$$

This means that, for every *i*, there exist two channels $D_{i},{\tilde{D}}_{i}\in \mathrm{Chan}\left(B\right)$ such that

(A43) $$\begin{array}{c} D_{i}\circ C_{i}={\tilde{D}}_{i}\circ C_{i+1}\;. \end{array}$$

Tracing over B on both sides, we obtain

(A44) $$\begin{array}{c} {\mathrm{Tr}}_{B}\circ \;D_{i}\circ C_{i}={\mathrm{Tr}}_{B}\circ \;{\tilde{D}}_{i}\circ C_{i+1}\;, \end{array}$$

and, using the no signalling condition of Lemma A5,

(A45) $$\begin{array}{c} {\mathrm{Tr}}_{B}\circ \;C_{i}={\mathrm{Tr}}_{B}\circ \;C_{i+1}\;. \end{array}$$

Since the above relation holds for every *i*, we obtained the equality ${\mathrm{Tr}}_{B}\circ \;C={\mathrm{Tr}}_{B}\circ \;C^{\prime }$. Conversely, suppose that ${\mathrm{Tr}}_{B}\circ \;C={\mathrm{Tr}}_{B}\circ \;C^{\prime }$. Then, Lemma A7 implies the equality

(A46) $$\begin{array}{c} A_{k}=A_{k}^{\prime }\;\forall k\;, \end{array}$$

where $A_{k}$ and $A_{k}^{\prime }$ are the quantum channels defined in Lemma A6. Now, let $D_{0}$ be the channel in Chan(B) defined in Equation (A32). By definition, we have

(A47) $$\begin{array}{cc} D_{0}\circ C & =\underset{k}{⨁}\;\left(I_{A_{k}}⊗\beta_{k}\;{\mathrm{Tr}}_{B_{k}}\right)\circ P_{k}\circ C\\ & =\underset{k}{⨁}\;\left(I_{A_{k}}⊗\beta_{k}\;{\mathrm{Tr}}_{B_{k}}\right)\circ \left(A_{k}⊗I_{B_{k}}\right)\circ P_{k}\\ & =\underset{k}{⨁}\;\left(A_{k}⊗\beta_{k}\;{\mathrm{Tr}}_{B_{k}}\right)\circ P_{k}\;. \end{array}$$

Similarly, we have

(A48) $$\begin{array}{c} D_{0}\circ C=\underset{k}{⨁}\;\left(A_{k}^{\prime }⊗\beta_{k}\;{\mathrm{Tr}}_{B_{k}}\right)\circ P_{k}\;. \end{array}$$

Since $A_{k}$ and $A_{k}^{\prime }$ are equal for every *k*, we conclude that $D_{0}\circ C$ is equal to $D_{0}\circ C^{\prime }$. This means that the intersection between Deg(C) and $\mathrm{Deg}(C^{\prime })$ is non-empty, and, therefore C is equivalent to $C^{\prime }$ modulo *B*. ☐

Combining Lemmas A7 and A8, we obtain the following corollary:

**Corollary** **A1.**
*For two channels $C,C^{\prime }\in \mathrm{Chan}\left(A\right)$, let $A_{k}$ and $A_{k}^{\prime }$ be the quantum channels defined in Lemma A6. Then, the following are equivalent:*

1. *C and $C^{\prime }$ are equivalent for A,*
2. *${⨁}_{k}A_{k}={⨁}_{k}A_{k}^{\prime }$.*

**Proof.** By Lemma A8, C and $C^{\prime }$ are equivalent for *A* if and only if the condition ${\mathrm{Tr}}_{B}\circ C={\mathrm{Tr}}_{B}\circ C^{\prime }$ holds. By Lemma A7, the condition ${\mathrm{Tr}}_{B}\circ C={\mathrm{Tr}}_{B}\circ C^{\prime }$ holds if and only if one has $A_{k}=A_{k}^{\prime }$ for every *k*. In turn, the latter condition holds if and only if the equality ${⨁}_{k}A_{k}={⨁}_{k}A_{k}^{\prime }$ holds. ☐

In summary, the transformations of system $S_{A}$ are characterized as

(A49) $$\begin{array}{c} \mathrm{Transf}\left(S_{A}\right)=\underset{k}{⨁}\;\mathrm{Chan}\left(A_{k}\right)\;, \end{array}$$

where $\mathrm{Chan}(A_{k})$ is the set of all quantum channels from $\mathrm{Lin}(H_{A_{k}})$ to itself.

To conclude, we observe that the transformations of $S_{A}$ act in the expected way. To this purpose, we consider the *restriction map*

(A50) $$\begin{array}{c} \pi_{A}:\mathrm{Chan}\left(A\right)\to \underset{k}{⨁}\;\mathrm{Chan}\left(A_{k}\right)\;,\;C\mapsto \underset{k}{⨁}\;A_{k}\;, \end{array}$$

where $A_{k}$ is defined as in Lemma A6.

Using the restriction map, we can prove the following propositions:

**Proposition** **A2.**
*For every channel C∈Chan(A), we have the relation*

(A51) $$\begin{array}{c} {\mathrm{Tr}}_{B}\circ \;C=\pi_{A}\left(C\right)\circ {\mathrm{Tr}}_{B}. \end{array}$$

*In words, evolving system S with C and then computing the local state of system $S_{A}$ is the same as computing the local state of system $S_{A}$ and then evolving it with $\pi_{A}\left(C\right)$.*

**Proof.** Using Lemma A6, the proof is straightforward:

(A52) $$\begin{array}{cc} {\mathrm{Tr}}_{B}\circ \;C & =\underset{k}{⨁}\;\left(I_{A_{k}}⊗{\mathrm{Tr}}_{B_{k}}\right)\circ P_{k}\circ C\\ & =\underset{k}{⨁}\;\left(I_{A_{k}}⊗{\mathrm{Tr}}_{B_{k}}\right)\circ \left(A_{k}⊗I_{B_{k}}\right)\circ P_{k}\\ & =\underset{k}{⨁}\;A_{k}\circ \left(I_{A_{k}}⊗{\mathrm{Tr}}_{B_{k}}\right)\circ P_{k}\\ & =\left(\underset{k}{⨁}\;A_{k}\right)\circ \left[\underset{l}{⨁}\left(I_{A_{l}}⊗{\mathrm{Tr}}_{B_{l}}\right)\circ P_{l}\right]\\ & =\pi_{A}\left(C\right)\circ {\mathrm{Tr}}_{B}\;. \end{array}$$

 ☐

**Proposition** **A3.**
*For every pair of channels $C_{1},C_{2}\in \mathrm{Chan}\left(A\right)$, we have the homomorphism relation*

(A53) $$\begin{array}{c} \pi_{A}\left(C_{1}\circ C_{2}\right)=\pi_{A}\left(C_{1}\right)\circ \pi_{A}\left(C_{2}\right)\;. \end{array}$$

**Proof.** Let us write the channels $\pi_{A}\left(C_{1}\right)$, $\pi_{A}\left(C_{2}\right)$, and $\pi_{A}\left(C_{1}\circ C_{2}\right)$ as

(A54) $$\begin{array}{c} \pi_{A}\left(C_{1}\right)=\underset{k}{⨁}\;A_{1k}\;,\;\pi_{A}\left(C_{2}\right)=\underset{k}{⨁}\;A_{2k}\;,\;\mathrm{and}\;\pi_{A}\left(C_{1}\circ C_{2}\right)=\underset{k}{⨁}\;A_{12k}\;. \end{array}$$

With this notation, we have

(A55) $$\begin{array}{cc} \left(A_{12k}⊗I_{B_{k}}\right)\circ P_{k} & =P_{k}\circ C_{1}\circ C_{2}\\ & =\left(A_{1k}⊗I_{B_{k}}\right)\circ P_{k}\circ C_{2}\\ & =\left(A_{1k}⊗I_{B_{k}}\right)\circ \left(A_{2k}⊗I_{B_{k}}\right)\circ P_{k}\\ & =\left[\left(A_{1k}\circ A_{2k}\right)⊗I_{B_{k}}\right]\circ P_{k}\;\forall k\;. \end{array}$$

From the above equation, we obtain the equality $A_{12k}=A_{1k}\circ A_{2k}$ for all *k*. In turn, this equality implies the desired result:

(A56) $$\begin{array}{cc} \pi_{A}\left(C_{1}\right)\circ \pi_{A}\left(C_{2}\right) & =\left(\underset{k}{⨁}A_{1k}\right)\circ \left(\underset{l}{⨁}A_{2l}\right)\\ & =\underset{k}{⨁}A_{1k}\circ A_{2k}\\ & =\underset{k}{⨁}A_{12k}\\ & =\pi_{A}\left(C_{1}\circ C_{2}\right)\;. \end{array}$$

 ☐

## Appendix D. Basis-Preserving and Multiphase-Covariant Channels

### Appendix D.1. Proof of Theorem 1

Here, we prove that the monoid of multiphase covariant channels on *S* (denoted as MultiPCov(S)) and the monoid of basis-preserving channels on *S* (denoted as BPres(S)) are one the commutant of the other.

The proof uses a few lemmas, the first of which is fairly straightforward:

**Lemma** **A9.**
*$\mathrm{BPres}{\left(S\right)}^{\prime }\subseteq \mathrm{MultiPCov}\left(S\right)$.*

**Proof.** Every unitary channel of the form $U_{\theta }=U_{\theta }\cdot U_{\theta }^{†}$ is basis-preserving, and therefore every channel C in the commutant of BPres(S) must commute with it. By definition, this means that C is multiphase covariant. ☐

To prove the converse inclusion, we use the following characterization of multiphase covariant channels:

**Lemma A10** (Characterization of MultiPCov(S))**.**
*A channel M∈Chan(S) is multiphase covariant if and only if it has a Kraus representation of the form*

(A57) $$\begin{array}{c} M\left(\rho \right)=\sum_{i=1}^{r}M_{i}\rho M_{i}^{†}+\sum_{k=1}^{d}\sum_{j\neq k}\;p\left(j|k\right)\;|j\rangle \langle k|\;\rho |k\rangle \langle j|\;, \end{array}$$

*where each operator $M_{i}$ is diagonal in the computational basis, and each p(j|k) is non-negative.*

**Proof.** Let $M\in \mathrm{Lin}(H_{S}⊗H_{S})$ be the Choi operator of channel M. For a multiphase covariant channel, the Choi operator must satisfy the commutation relation [87,88]

(A58) $$\begin{array}{c} \left[M,U_{\theta }⊗{\bar{U}}_{\theta }\right]=0\;\forall \theta \in {\left[0,2\pi \right)}^{⊗d}\;. \end{array}$$

This condition implies that *M* must have the form

(A59) $$\begin{array}{c} M=\sum_{s,t}M_{ss,tt}\;|s\rangle \langle t|⊗\left|s\rangle \langle t\right|+\sum_{k}\sum_{j\neq k}\;M_{jk,jk}\;|j\rangle \langle j|⊗|k\rangle \langle k|\;, \end{array}$$

where the d×d matrix $\left[\Gamma_{s,t}\right]:={\left[M_{ss,tt}\right]}_{s,t\in \{1,\cdots ,d\}}$ is positive semidefinite and each coefficient $M_{st,st}$ is non-negative. Then, Equation (A57) follows from diagonalizing the matrix Γ and using the relation $M\left(\rho \right)=\mathrm{Tr}[M\;\left(I⊗\rho^{T}\right)]$, where $\rho^{T}$ is the transpose of ρ in the computational basis. ☐

From Equation (A57), one can show every multiphase covariant channel commutes with every basis-preserving channel:

**Lemma** **A11.**
*$\mathrm{MultiPCov}\left(S\right)\subseteq \mathrm{BPres}{\left(S\right)}^{\prime }$.*

**Proof.** Let B∈BPres(S) be a generic basis-preserving channel, and let M∈MultiPCov(S) be a generic multiphase covariant channel. Using the characterization of Equation (A57), we obtain

(A60) $$\begin{array}{cc} M\circ B(\rho ) & =\sum_{i}\;M_{i}B\left(\rho \right)M_{i}^{†}+\sum_{k}\sum_{j\neq k}\;p\left(j|k\right)|j\rangle \langle k|B\left(\rho \right)|k\rangle \langle j|\\ & =\sum_{i}\;B\left(M_{i}\rho M_{i}^{†}\right)+\sum_{k}\sum_{j\neq k}\;p\left(j|k\right)|j\rangle \langle k|B\left(\rho \right)\left|k\rangle \langle j\right|\\ & =\sum_{i}\;B\left(M_{i}\rho M_{i}^{†}\right)+\sum_{k}\sum_{j\neq k}\;p\left(j|k\right)\left|j\rangle \;\langle k|\rho |k\rangle \;\langle j\right|\\ & =\sum_{i}\;B\left(M_{i}\rho M_{i}^{†}\right)+\sum_{k}\sum_{j\neq k}\;p\left(j|k\right)\;B(|j\rangle \langle j|)\;\langle k|\rho |k\rangle \;\\ & =B\left(\sum_{i}M_{i}\rho M_{i}^{†}+\sum_{k}\sum_{j\neq k}\;p\left(j|k\right)|j\rangle \;\langle k|\rho |k\rangle \;\langle j|\right)\\ & =B\circ M(\rho )\;\forall \rho \in \mathrm{Lin}(S)\;. \end{array}$$

The second equality used the fact that the Kraus operators of B are diagonal in the computational basis [71,72] and therefore commute with each operator $M_{i}$. The third equality uses the relation 〈k|B(ρ)|k〉=〈k|ρ|k〉, following from the fact that B preserves the computational basis [71,72]. ☐

Summarizing, we have shown that the multiphase covariant channels are the commutant of the basis-preserving channels:

**Corollary** **A2.**
*$\mathrm{MultiPCov}\left(S\right)=\mathrm{BPres}{\left(S\right)}^{\prime }$.*

Note that Corollary A2 implies the relation

(A61) $$\begin{array}{c} \mathrm{MultiPCov}{\left(S\right)}^{\prime }=\mathrm{BPres}{\left(S\right)}^{\prime \prime }⊇\mathrm{BPres}\left(S\right)\;. \end{array}$$

To conclude the proof of Theorem 1, we prove the converse inclusion:

**Lemma** **A12.**
*$\mathrm{MultiPCov}{\left(S\right)}^{\prime }\subseteq \mathrm{BPres}\left(S\right)$.*

**Proof.** A special case of multiphase covariant channel is the erasure channel $M_{k}$ defined by $M_{k}\left(\rho \right)=\left|k\rangle \langle k\right|$ for every ρ∈Lin(S). For a generic channel $C\in \mathrm{MultiPCov}{\left(S\right)}^{\prime }$, one must have

(A62) $$\begin{array}{c} C(|k\rangle \langle k|)=C\circ M_{k}(|k\rangle \langle k|)=M_{k}\circ C(|k\rangle \langle k|)=|k\rangle \langle k|\;. \end{array}$$

Since the above condition must hold for every *k*, the channel C must be basis-preserving. ☐

Combining Lemma A12 and Equation (A61), we obtain:

**Corollary** **A3.**
*$\mathrm{MultiPCov}{\left(S\right)}^{\prime }=\mathrm{BPres}\left(S\right)$.*

Putting Corollaries A2 and A3 together, we have an immediate proof of Theorem 1.

### Appendix D.2. Proof of Equation (55)

Here, we show that the transformations on system $S_{A}$ are classical channels. To construct the transformations of $S_{A}$, we have to partition the double commutant of Act(A;S)=MultiPCov(S) into equivalence classes.

First, recall that $\mathrm{MultiPCov}{\left(S\right)}^{\prime \prime }=\mathrm{MultiPCov}\left(S\right)$ (by Theorem 1). Then, note the following property:

**Lemma** **A13.**
*If two channels $M,\tilde{M}\in \mathrm{MultiPCov}\left(S\right)$ satisfy the condition*

(A63) $$\begin{array}{c} \langle k|\;M(|j\rangle \langle j|)\;|k\rangle =\langle k|\;\tilde{M}(|j\rangle \langle j|)\;|k\rangle \;, \end{array}$$

*then ${\left[M\right]}_{A^{\prime }}={\left[\tilde{M}\right]}_{A^{\prime }}$.*

**Proof.** Define the completely dephasing channel $D=\sum_{k}\;|k\rangle \langle k|\cdot \left|k\rangle \langle k\right|$. Clearly, D is basis-preserving. Using the idempotence relation D∘D=D, we obtain

(A64) $$\begin{array}{cc} \left(D\circ M\right)\;\left(\rho \right) & =\left(D\circ D\circ M\right)\;\left(\rho \right)\\ & =\left(D\circ M\circ D\right)\;\left(\rho \right)\\ & =\left(D\circ M\right)\;\left(\sum_{j}\;|j\rangle \langle j|\;\langle j|\rho |j\rangle \right)\\ & =\sum_{j}\;\langle j|\rho |j\rangle \;D\;\left(M(|j\rangle \langle j|)\right)\\ & =\sum_{j,k}\;\langle j|\rho |j\rangle \;\langle k|M(|j\rangle \langle j|)|k\rangle \;|k\rangle \langle k|\;. \end{array}$$

Likewise, we have

(A65) $$\begin{array}{c} \left(D\circ \tilde{M}\right)\;\left(\rho \right)=\sum_{j,k}\;\langle j|\rho |j\rangle \;\langle k|\tilde{M}(|j\rangle \langle j|)|k\rangle \;|k\rangle \langle k|\;. \end{array}$$

If condition (A63) holds, then the equality $D\circ M=D\circ \tilde{M}$ holds, meaning that Deg(M) and $\mathrm{Deg}(\tilde{M})$ have non-empty intersection. Hence, M and $\tilde{M}$ must be in the same equivalence class. ☐

The converse of Lemma A13 holds:

**Lemma** **A14.**
*If two channels $M,\tilde{M}\in \mathrm{MultiPCov}\left(S\right)$ are in the same equivalence class, then they must satisfy condition (A63).*

**Proof.** If M and $\tilde{M}$ are in the same equivalence class, then there exists a finite sequence $\left(M_{1},M_{2},\cdots ,M_{n}\right)$ such that

$$\begin{array}{c} M_{1}=M\;,\;M_{n}=\tilde{M}\;,\;\forall i\in \left\{1,\cdots ,n-1\right\}\;\exists B_{i},\tilde{B_{i}}\in \mathrm{BPres}\left(S\right):\;B_{i}\circ M_{i}=\tilde{B_{i}}\circ M_{i+1}\;. \end{array}$$

The above condition implies

(A66) $$\begin{array}{c} l\langle k|\;M_{i}\left(\rho \right)\;|k\rangle =\mathrm{Tr}[M_{i}\left(\rho \right)\;|k\rangle \langle k|]=\langle k|\;B_{i}\circ M_{i}\left(\rho \right)\;|k\rangle =\langle k|\;\tilde{B_{i}}\circ M_{i+1}\left(\rho \right)\;|k\rangle \\ =\langle k|\;M_{i+1}\left(\rho \right)\;|k\rangle \;, \end{array}$$

for all i∈{1,⋯,n−1} and for all ρ∈Lin(ρ). In particular, choosing ρ=|j〉〈j| we obtain

(A67) $$\begin{array}{c} \langle k|\;M_{i}(|j\rangle \langle j|)\;|k\rangle =\langle k|\;M_{i+1}(|j\rangle \langle j|)\;|k\rangle \;\forall i\in \left\{1,\cdots ,n-1\right\}\;,\forall j,k\in \left\{1,\cdots ,d\right\}\;. \end{array}$$

Hence, Equation (A63) follows. ☐

## Appendix E. Classical Systems and the Resource Theory of Coherence

Here, we consider agents who have access to various types of free operations in the resource theory of coherence. We start from the types of operations that give rise to classical systems, and then show two examples that do not have this property.

### Appendix E.1. Operations That Lead to Classical Subsystems

Consider the following monoids of operations

1. *Strictly incoherent operations [41],* i.e., quantum channels T with the property that, for every Kraus operator $T_{i}$, the map $T_{i}\left(\cdot \right)=T_{i}\cdot T_{i}$ satisfies the condition $D\circ T_{i}=T_{i}\circ D$, where D is the completely dephasing channel.
2. *Dephasing covariant operations* [38,39,40], i.e., quantum channels T satisfying the condition D∘T=T∘D.
3. *Phase covariant channels* [40], i.e., quantum channels T satisfying the condition $T\circ U_{\varphi }=U_{\varphi }\circ T$, ∀φ∈[0,2π), where $U_{\varphi }$ is the unitary channel associated with the unitary matrix $U_{\varphi }=\sum_{k}\;e^{ik\varphi }\;|k\rangle \langle k|$.
4. *Physically incoherent operations* [38,39], i.e., quantum channels that are convex combinations of channels T admitting a Kraus representation where each Kraus operator $T_{i}$ is of the form
  (A68) $$\begin{array}{c} T_{i}=U_{\pi_{i}}\;U_{\theta_{i}}\;P_{i}\;, \end{array}$$
  where $U_{\pi_{i}}$ is a unitary that permutes the elements of the computational basis, $U_{\theta_{i}}$ is a diagonal unitary, and $P_{i}$ is a projector on a subspace spanned by a subset of vectors in the computational basis.
5. *Classical channels* i.e., channels satisfying T=D∘T∘D.

We now show that all the above operations define classical subsystems according to our construction.

The first ingredient in the proof is the observation that each of the monoids 1–5 contains the monoid of classical channels. Then, we can apply the following lemma:

**Lemma** **A15.**
*Let M⊆Chan(S) be a monoid of quantum channels, and let $M^{\prime }$ be its commutant. If M contains the monoid of classical channels, then $M^{\prime }$ is contained in the set of basis-preserving channels.*

**Proof.** Consider the erasure channel $C_{k}$ defined by $C_{k}\left(\rho \right):=|k\rangle \langle k|\;\mathrm{Tr}\left[\rho \right]$, $\forall \rho \in \mathrm{Lin}(H_{S})$. Clearly, the erasure channel is a classical channel. Then, every channel $B\in M^{\prime }$ must satisfy the condition

(A69) $$\begin{array}{c} B(|k\rangle \langle k|)=B\circ C_{k}(|k\rangle \langle k|)=C_{k}\circ B(|k\rangle \langle k|)=|k\rangle \langle k|\;. \end{array}$$

Since *k* is generic, this implies that B must be basis-preserving. ☐

Furthermore, we have the following

**Lemma** **A16.**
*Let Act(A;S)⊆Chan(S) be a set of quantum channels that contains the monoid of classical channels. If two quantum states ρ,σ∈St(S) are equivalent for A, then they must have the same diagonal entries. Equivalently, they must satisfy D(ρ)=D(σ).*

**Proof.** Same as the first part of the proof of Proposition 7. Suppose that Condition 1 holds, meaning that there exists a sequence $\left(\rho_{1},\rho_{2},\cdots ,\rho_{n}\right)$ such that

(A70) $$\begin{array}{c} \rho_{1}=\rho \;,\;\rho_{n}=\sigma \;,\;\forall i\in \left\{1,\cdots ,n-1\right\}\;\exists B_{i}\;,\tilde{B_{i}}\in \mathrm{Act}\left(B;S\right):\;B_{i}\left(\rho_{i}\right)=\tilde{B_{i}}\left(\rho_{i+1}\right)\;, \end{array}$$

where $B_{i}$ and $\tilde{B_{i}}$ are channels in the commutant $\mathrm{Act}{\left(A;S\right)}^{\prime }$. The above equation implies

(A71) $$\begin{array}{c} \langle k|B_{i}\left(\rho_{i}\right)|k\rangle =\langle k|\tilde{B_{i}}\left(\rho_{i+1}\right)|k\rangle \;. \end{array}$$

Now, we know that the commutant $\mathrm{Act}{\left(A;S\right)}^{\prime }$ consists of basis-preserving channels (Lemma A15). Since every basis-preserving channel satisfies the relation 〈k|B(ρ)|k〉=〈k|ρ|k〉 [71,72], we obtain that all the density matrices $\left(\rho_{1},\rho_{2},\cdots ,\rho_{n}\right)$ must have the same diagonal entries, namely $D\left(\rho_{1}\right)=D\left(\rho_{2}\right)=\cdots =D\left(\rho_{n}\right)$. ☐

Now, we observe that the completely dephasing channel D is contained in the commutant of all the monoids 1–5. This fact is evident for the monoids 1, 2 and 5, where the commutation with D holds by definition. For the monoid 3, the commutation with D has been proven in [38,39], and for the monoid 4 it has been proven in [40].

Since D is contained in the commutant of all the monoids 1–5, we can use the following obvious fact:

**Lemma** **A17.**
*Let Act(A;S)⊆Chan(S) be a monoid of quantum channels and suppose that its commutant $\mathrm{Act}{\left(A;S\right)}^{\prime }$ contains the dephasing channel D. If two quantum states ρ,σ∈St(S) satisfy D(ρ)=D(σ), then they are equivalent for A.*

**Proof.** Trivial consequence of the definition. ☐

Combining Lemmas A16 and A17, we obtain the following

**Proposition** **A4.**
*Let Act(A;S)⊆Chan(S) be a monoid of quantum channels on system S. If Act(A;S) contains the monoid of classical channels, and if the the commutant $\mathrm{Act}{\left(A;S\right)}^{\prime }$ contains the completely dephasing channel D, then two states ρ,σ∈St(S) are equivalent for A if and only if D(ρ)=D(σ).*

**Proof.** Same as the proof of Proposition 7. ☐

Proposition A4 implies that the states of the subsystem $S_{A}$ are in one-to-one correspondence with diagonal density matrices. Since the conditions of the proposition are satisfied by all the monoids 1–5, each of these monoids defines the same state space.

The same result holds for the transformations:

**Proposition** **A5.**
*Let Act(A;S)⊆Chan(S) be a monoid of quantum channels. If Act(A;S) contains the monoid of classical channels, and if the the commutant $\mathrm{Act}{\left(A;S\right)}^{\prime }$ contains the completely dephasing channel D, then two transformations S,T∈Transf(S) are equivalent for A if and only if D∘T∘D=D∘T∘D.*

**Proof.** Same as the proofs of Lemmas A13 and A14. ☐

Proposition A5 implies that the transformations of subsystem $S_{A}$ can be identified with classical channels. Hence, system $S_{A}$ is exactly the *d*-dimensional classical subsystem of the quantum system *S*. In summary, each of the monoids 1–5 defines the same *d*-dimensional classical subsystem.

### Appendix E.2. Operations That Do Not Lead to Classical Subsystems

Here, we show that our construction does not associate classical subsystems with the monoids of incoherent and maximally incoherent operations. To start with, we recall the definitions of these two subsets:

1. The *maximally incoherent operations* are the quantum channels T that map diagonal density matrices to diagonal density matrices, namely T∘D=D∘T∘D, where D is the completely dephasing channel.
2. The *Incoherent operations* are the quantum channels T with the property that, for every Kraus operator $T_{i}$, the map $T_{i}\left(\cdot \right)=T_{i}\cdot T_{i}$ sends diagonal matrices to diagonal matrices, namely $T_{i}\circ D=D\circ T_{i}\circ D$.

Note that each set of operations contains the set of classical channels. Hence, the commutant of each set of operation consists of (some subset of) basis-preserving channels (by Lemma A15).

Moreover, both sets of operations 1 and 2 contain the set of quantum channels $C_{\psi }$ defined by the relation

(A72) $$\begin{array}{c} C_{\psi }\left(\rho \right)=|1\rangle \langle 1|\;\langle \psi |\rho |\psi \rangle +\frac{I-|1\rangle \langle 1|}{d-1}\;\mathrm{Tr}\left[(I-|\psi \rangle \langle \psi |)\;\rho \right]\;\forall \rho \in \mathrm{Lin}\left(H_{S}\right)\;, \end{array}$$

where $|\psi \rangle \in H_{S}$ is a fixed (but otherwise arbitrary) unit vector. The fact that both monoids contain the channels $C_{\psi }$ implies a strong constraint on their commutants:

**Lemma** **A18.**
*The only basis-preserving quantum quantum channel B∈BPres(S) satisfying the property $B\circ C_{\psi }=C_{\psi }\circ B$ for every $|\psi \rangle \in H_{S}$ is the identity channel.*

**Proof.** The commutation property implies the relation

(A73) $$\begin{array}{cc} \left(C_{\psi }\circ B\right)\;(|\psi \rangle \langle \psi |) & =\left(B\circ C_{\psi }\right)\;(|\psi \rangle \langle \psi |)\\ & =B(|1\rangle \langle 1|)\\ & =|1\rangle \langle 1|\;, \end{array}$$

where we used the fact that B is basis-preserving. Tracing both sides of the equality with the projector |1〉〈1|, we obtain the relation

(A74) $$\begin{array}{cc} 1 & =\langle 1|\left(C_{\psi }\circ B\right)\;(|\psi \rangle \langle \psi |)|1\rangle \\ & =\langle \psi |\;B(|\psi \rangle \langle \psi |)\;|\psi \rangle \;, \end{array}$$

the second equality following from the definition of channel $C_{\psi }$. In turn, Equation (A74) implies the relation B(|ψ〉〈ψ|)=|ψ〉〈ψ|. Since |ψ〉 is arbitrary, this means that B must be the identity channel. ☐

In summary, the commutant of the set of incoherent channels consists only of the identity channel, and so is the the commutant of the set of maximally incoherent channels. Since the commutant is trivial, the equivalence classes are trivial, meaning that the subsystem $S_{A}$ has exactly the same states and the same transformations of the original system *S*. In short, the subsystem associated with the incoherent (or maximally incoherent) channels is the full quantum system.

## Appendix F. Enriching the Sets of Transformations

Here, we provide a mathematical construction that enlarges the sets of transformations in the “baby category” with objects $S,S_{A},$ and $S_{B}$. This construction provides a realization of a catagorical structure known as splitting of idempotents [73,74].

As we have seen in the main text, our basic construction does not provide transformations from the subsystem $S_{A}$ to the global system *S*. One could introduce such transformations by hand, by defining an *embedding* [63]:

**Definition** **A1.** *An* embedding *of $S_{A}$ into S is a map $E_{A}:\mathrm{St}\left(S_{A}\right)\to \mathrm{St}\left(S\right)$ satisfying the property*

(A75) $$\begin{array}{c} {\mathrm{Tr}}_{B}\circ E_{A}=I_{S_{A}}\;. \end{array}$$

*In other words, $E_{A}$ associates a representative to every equivalence class $\rho \in \mathrm{St}(S_{A})$.*

A priori, embeddings need not be physical processes. Consider the example of a classical system, viewed as a subsystem of a closed quantum system as in Section 4.3. An embedding would map each classical probability distribution $\left(p_{1},p_{2},\cdots ,p_{d}\right)$ into a pure quantum state $|\psi \rangle =\sum_{k}\;c_{k}\;|k\rangle$ satisfying the condition $|c_{k}{|}^{2}=p_{k}$ for all k∈{1,⋯,d}. If the embedding were a physical transformation, there would be a way to physically transform every classical probability distributions into a corresponding pure quantum state, a fact that is impossible in standard quantum theory.

When building a new physical theory, one could *postulate* that there exists an embedding $E_{A}$ that is physically realizable. In that case, the transformations from $S_{A}$ to *S* would be those in the set

(A76) $$\begin{array}{c} \mathrm{Transf}\left(S_{A}\to S\right)=\left\{T\circ E_{A}\;:\;T\in \mathrm{Transf}\left(S\right)\right\}\;, \end{array}$$

and similarly for the transformations from $S_{B}$ to *S*. The transformations from $S_{A}$ to $S_{B}$ would be those in the set

(A77) $$\begin{array}{c} \mathrm{Transf}\left(S_{A}\to S_{B}\right)=\left\{{\mathrm{Tr}}_{A}\circ T\circ E_{A}\;:\;T\in \mathrm{Transf}\left(S\right)\right\}\;, \end{array}$$

and similarly for the transformations from $S_{B}$ to $S_{A}$. In that new theory, the old set of transformations from $S_{A}$ should be replaced by the new set:

(A78) $$\begin{array}{c} \tilde{\mathrm{Transf}}\left(S_{A}\right)=\left\{{\mathrm{Tr}}_{B}\circ T\circ E_{A}\;:\;T\in \mathrm{Transf}\left(S\right)\right\}\;, \end{array}$$

so that the structure of category is preserved. Similarly, the old set of transformations from $S_{B}$ to $S_{B}$ should be replaced by the new set.

(A79) $$\begin{array}{c} \tilde{\mathrm{Transf}}\left(S_{B}\right)=\left\{{\mathrm{Tr}}_{A}\circ T\circ E_{B}\;:\;T\in \mathrm{Transf}\left(S\right)\right\}\;. \end{array}$$

When this is done, the embeddings define two idempotent morphisms $P_{A}:=E_{A}\circ {\mathrm{Tr}}_{B}$ and $P_{B}:=E_{B}\circ {\mathrm{Tr}}_{A}$, i.e., two morphisms satisfying the conditions

(A80) $$\begin{array}{c} P_{A}\circ P_{A}=P_{A}\;\mathrm{and}\;P_{B}\circ P_{B}=P_{B}\;. \end{array}$$

The partial trace and the embedding define a *splitting of idempotents*, in the sense of Refs. [73,74]. The splitting of idempotents was considered in the categorical framework as a way to define general decoherence maps, and, more specifically, decoherence maps to classical subsystems [74,89].

## Appendix G. The Total System as a Subsystem

For every system satisfying the Non-Overlapping Agents Requirement, the system *S* can be regarded as a subsystem:

**Proposition** **A6.**
*Let S be a system satisfying the Non-Overlapping Agents Requirement, let $A_{\max}$ be the maximal agent, and $S_{A_{\max}}$ be the associated subsystem. Then, one has $S_{A_{\max}}≃S$, meaning that there exist two isomorphisms $\gamma :\mathrm{St}\left(S\right)\to \mathrm{St}\left(S_{A_{\max}}\right)$ and $\delta :\mathrm{Transf}\left(S\right)\to \mathrm{Transf}\left(S_{A_{\max}}\right)$ satisfying the condition*

(A81) $$\begin{array}{c} \gamma (T\psi )=\delta (T)\;\gamma (\psi )\;,\;\forall \psi \in \mathrm{St}(S)\;,\forall T\in \mathrm{Transf}(S)\;. \end{array}$$

**Proof.** The Non-Overlapping Agents Requirement guarantees that the commutant $\mathrm{Act}{\left(A_{\max};S\right)}^{\prime }$ contains only the identity transformation. Hence, the equivalence class ${\left[\psi \right]}_{A_{\max}}$ contains only the state ψ. Hence, the partial trace ${\mathrm{Tr}}_{A_{\max}^{\prime }}:\psi \mapsto {\left[\psi \right]}_{A_{\max}}$ is a bijection from St(S) to $\mathrm{St}\left(S_{A_{\max}}\right)$. Similarly, the equivalence class ${\left[T\right]}_{A_{\max}}$ contains only the transformation T. Hence, the restriction $\pi_{A_{\max}}:T\mapsto {\left[T\right]}_{A_{\max}}$ is a bijective function between Transf(S) and $\mathrm{Transf}\left(S_{A_{\max}}\right)$. Such a function is an homomorphism of monoids, by Equation (20). Setting $\delta :=\pi_{A_{\max}}$ and $\gamma :={\mathrm{Tr}}_{A_{\max}^{\prime }}$, the condition (A81) is guaranteed by Equation (21). ☐

## Appendix H. Proof of Proposition 15

By definition, the condition ${\mathrm{Tr}}_{B}\left[\psi \right]={\mathrm{Tr}}_{B}\left[\psi^{\prime }\right]$ holds if and only if there exists a finite sequence $\left(\psi_{1},\psi_{2},\cdots ,\psi_{n}\right)$ such that

(A82) $$\begin{array}{c} \psi_{1}=\psi \;,\;\psi_{n}=\psi^{\prime }\;,\;\forall i\in \left\{1,\cdots ,n-1\right\}\;\exists V_{i}\;,\tilde{V_{i}}\in \mathrm{Act}\left(B;S\right):\;V_{i}\psi_{i}={\tilde{V}}_{i}\psi_{i+1}\;.\; \end{array}$$

Our goal is to prove that there exists an adversarial action $V_{B}\in \mathrm{Act}\left(B;S\right)$ such that the relation $\psi^{\prime }=V_{B}\psi$ or $\psi =V_{B}\psi^{\prime }$ holds.

We will proceed by induction on *n*, starting from the base case n=2. In this case, we have ${\mathrm{Deg}}_{B}\left(\psi \right)\cap {\mathrm{Deg}}_{B}\left(\psi^{\prime }\right)\neq \emptyset$. Then, the first regularity condition implies that there exists a transformation $V_{B}\in \mathrm{Act}\left(B;S\right)$ such that at least one of the relations $V_{B}\psi =\psi^{\prime }$ and $\psi =V_{B}\psi^{\prime }$ holds. This proves the validity of the base case.

Now, suppose that the induction hypothesis holds for all sequences of length *n*, and suppose that ψ and $\psi^{\prime }$ are equivalent through a sequence of length n+1, say $\left(\psi_{1},\psi_{2},\cdots ,\psi_{n},\psi_{n+1}\right)$. Applying the induction hypothesis to the sequence $\left(\psi_{1},\psi_{2},\cdots ,\psi_{n}\right)$, we obtain that there exists a transformation V∈Act(B;S) such that at least one of the relations $\psi_{n}=V\psi$ and $\psi =V\psi_{n}$ holds. Moreover, applying the induction hypothesis to the pair $\left(\psi_{n},\psi_{n+1}\right)$ we obtain that there exists a transformation $V^{\prime }\in \mathrm{Act}\left(B;S\right)$ such that $\psi_{n+1}=V^{\prime }\psi_{n}$, or $\psi_{n}=V^{\prime }\psi_{n+1}$. Hence, there are four possible cases:

1. $\psi_{n}=V\psi$ and $\psi_{n+1}=V^{\prime }\psi_{n}$. In this case, we have $\psi_{n+1}=\left(V^{\prime }\circ V\right)\psi$, which proves the desired statement.
2. $\psi_{n}=V\psi$ and $\psi_{n}=V^{\prime }\psi_{n+1}$. In this case, we have $V\psi =V^{\prime }\psi_{n+1}$, or equivalently ${\mathrm{Deg}}_{B}\left(\psi \right)\cap {\mathrm{Deg}}_{B}\left(\psi_{n+1}\right)\neq \emptyset$. Applying the induction hypothesis to the sequence $\left(\psi ,\psi_{n+1}\right)$, we obtain the desired statement.
3. $\psi =V\psi_{n}$ and $\psi_{n+1}=V^{\prime }\psi_{n}$. Using the second regularity condition, we obtain that there exists a transformation W∈Act(B;S) such that at least one of the relations $V=W\circ V^{\prime }$ and $V^{\prime }=W\circ V$ holds. Suppose that $V=W\circ V^{\prime }$. In this case, we have
  (A83) $$\begin{array}{c} \psi =V\psi_{n}=\left(W\circ V^{\prime }\right)\psi_{n}=W\psi_{n+1}\;. \end{array}$$
  Alternatively, suppose that $V^{\prime }=W\circ V$. In this case, we have
  (A84) $$\begin{array}{c} \psi_{n+1}=V^{\prime }\psi_{n}=\left(W\circ V\right)\psi_{n}=W\psi \;. \end{array}$$
  In both cases, we proved the desired statement.
4. $\psi =V\psi_{n}$ and $\psi_{n}=V^{\prime }\psi_{n+1}$. In this case, we have $\psi =\left(V\circ V^{\prime }\right)\psi_{n+1}$, which proves the desired statement.

☐

## Appendix I. Characterization of the Adversarial Group

Here, we provide the proof of Theorem 3, proving a canonical decomposition of the elements of the adversarial group. The proof proceeds in a few steps:

**Lemma** **A19** (Canonical form of the elements of the adversarial group)**.**
*Let $U:g\mapsto U_{g}$ be a projective representation of the group G, let Irr(U) be the set of irreducible representations contained in the isotypic decomposition of U, and let ω:G→C be a multiplicative character of G. Then, the commutation relation*

(A85) $$\begin{array}{c} VU_{g}=\omega \left(g\right)\;U_{g}V\;\forall g\in G \end{array}$$

*holds iff*

1. *The map $U^{\left(j\right)}\mapsto \omega \;U^{\left(j\right)}$ is a permutation of the set Irr(U), denoted as π:Irr(U)→Irr(U). In other words, for every irrep $U^{\left(j\right)}$ with j∈Irr(U), the irrep $\omega \;U^{\left(j\right)}$ is equivalent to an irrep k∈Irr(U), and the correspondence between j and k is bijective.*
2. *The multiplicity spaces $M_{j}$ and $M_{\pi (j)}$ have the same dimension.*
3. *The unitary operator V has the canonical form $V=U_{\pi }V_{0}$, where $V_{0}$ is an unitary operator in the commutant $U^{\prime }$ and $U_{\pi }$ is a permutation operator satisfying*
  (A86) $$\begin{array}{c} U_{\pi }\left(R_{j}⊗M_{j}\right)=\left(R_{\pi (j)}⊗M_{\pi (j)}\right)\;\forall j\in \mathrm{Irr}\left(U\right)\;. \end{array}$$

**Proof.** Let us use the isotypic decomposition of *U*, as in Equation (88). We define

(A87) $$\begin{array}{c} V_{j,k}:=\Pi_{j}\;V\;\Pi_{k}\;, \end{array}$$

where $\Pi_{j}$ ($\Pi_{k}$) is the projector onto $R_{j}⊗M_{j}$ ($R_{k}⊗M_{k}$). Then, Equation (A85) is equivalent to the condition

(A88) $$\begin{array}{c} V_{j,k}\;\left(U_{g}^{\left(k\right)}⊗I_{M_{k}}\right)=\omega \left(g\right)\;\left(U_{g}^{\left(j\right)}⊗I_{M_{j}}\right)\;V_{jk}\;,\;\forall g\in G\;,\forall j,k\;, \end{array}$$

which in turn is equivalent to the condition

(A89) $$\begin{array}{c} \langle \alpha |V_{j,k}|\beta \rangle \;\;U_{g}^{\left(k\right)}=\omega \left(g\right)\;U_{g}^{\left(j\right)}\;\langle \alpha |V_{j,k}|\beta \rangle \;,\;\forall g\in G\;,\forall j,k\;,\forall |\alpha \rangle \in M_{j}\;,\forall |\beta \rangle \in M_{k}\;, \end{array}$$

where $\langle \alpha |V_{j,k}|\beta \rangle$ is a shorthand for the partial matrix element $\left(I_{R_{j}}⊗\langle \alpha |)\;V_{j,k}\;(I_{R_{k}}⊗|\beta \rangle \right)$. Equation (A89) means that each operator $\langle \alpha |V_{j,k}|\beta \rangle$ intertwines the two representations $U^{\left(k\right)}$ and $\omega \;U^{\left(j\right)}$. Recall that each representation is irreducible. Hence, the second Schur’s lemma [78] implies that $\langle \alpha |\;V_{j,k}\;|\beta \rangle$ is zero if the two representations are not equivalent. Note that there can be *at most* one value of *j* such that $U^{\left(k\right)}$ is equivalent to $\omega \;U^{\left(j\right)}$. If such a value exists, we denote it as j=π(k). By construction, the function π:Irr(U)→Irr(U) must be injective. When j=π(k), the first Schur’s lemma [78] guarantees that the operator $\langle \alpha |\;V_{\pi (k),k}|\beta \rangle$ is proportional to the partial isometry $T_{\pi (k),k}$ that implements the equivalence of the two representations. Let us write

(A90) $$\begin{array}{c} \langle \alpha |\;V_{\pi (k),k}\;|\beta \rangle =M_{\alpha ,\beta }\;T_{\pi (k),k}\;, \end{array}$$

for some $M_{\alpha ,\beta }^{\left(k\right)}\in C$. Note also that, since the left-hand side is sesquilinear in |α〉 and |β〉, the right-hand side should also be sesquilinear. Hence, we can find an operator $M_{\pi (k),k}:M_{k}\to M_{\pi (k)}$ such that $M_{\alpha ,\beta }^{\left(k\right)}=\langle \alpha |\;M_{\pi (k),k}\;|\beta \rangle$. Putting everything together, the operator *V* can be written as

(A91) $$\begin{array}{c} V=\underset{k\in \mathrm{Irr}(U)}{⨁}\;\left(T_{\pi (k),k}⊗M_{\pi (k),k}\right)\;. \end{array}$$

Now, the operator *V* must be unitary, and, in particular, it should satisfy the condition $VV^{†}=I$, which reads

(A92) $$\begin{array}{c} \underset{k\in \mathrm{Irr}(U)}{⨁}\left(I_{R_{\pi (k)}}⊗M_{\pi (k),k}M_{\pi (k),k}^{†}\right)=I\;. \end{array}$$

The above condition implies that: (i) the function π must be surjective, and (ii) the operator $M_{\pi (k),k}$ must be a co-isometry. From the relation $V^{†}V$, we also obtain that $M_{\pi (k),k}$ must be an isometry. Hence, $M_{\pi (k)}$ is unitary. Summarizing, the condition (A85) can be satisfied only if there exists a permutation π:Irr(U)→Irr(U) such that, for every *j*,

1. the irreps $\omega \;U^{\left(k\right)}$ and $U^{\pi (k)}$ are equivalent,
2. the multiplicity spaces $M_{k}$ and $M_{\pi (k)}$ are unitarily isomorphic.

Fixing a unitary isomorphism $S_{\pi (k),k}:M_{k}\to M_{\pi (k)}$, we can write every element of the adversarial group in the canonical form $V=U_{\pi }\;V_{0}$, where $U_{\pi }$ is the permutation operator

(A93) $$\begin{array}{c} U_{\pi }=\underset{k\in \mathrm{Irr}(U)}{⨁}\;\left(T_{\pi (k),k}⊗S_{\pi (k),k}\right)\;, \end{array}$$

and $V_{0}$ is an element of the commutant $U^{\prime }$, i.e., a generic unitary operator of the form

(A94) $$\begin{array}{c} V_{0}=\underset{k\in \mathrm{Irr}(U)}{⨁}\;\left(I_{j}⊗V_{0,k}\right)\;. \end{array}$$

Conversely, if a permutation π exists with the properties that for every k∈Irr(U)

1. $\omega \;U^{\left(k\right)}$ and $U^{\left(\pi (k)\right)}$ are equivalent irreps,
2. $M_{k}$ and $M_{\pi (k)}$ are unitarily equivalent,

and if the operator *V* has the form $V=U_{\pi }V_{0}$, with $U_{\pi }$ and $V_{0}$ as in Equations (A93) and (A94), then *V* satisfies the commutation relation (A85). ☐

We have seen that every element of the adversarial group can be decomposed into the product of a permutation operator, which permutes the irreps, and an operator in the commutant of the original group representation U:G→Lin(H). We now observe that the allowed permutations have an additional structure: they must form an Abelian group, denoted as A.

**Lemma** **A20.**
*The permutations π arising from Equation (A85) with a generic multiplicative character ω(V,·) form an Abelian subgroup A of the group of all permutations of Irr(U).*

**Proof.** Let *V* and *W* be two elements of the adversarial group $G_{B}$, let ω(V,·) and ω(W,·) be the corresponding characters, and let $\pi_{V}$ and $\pi_{W}$ be the permutations associated with ω(V,·) and ω(W,·) as in Theorem A19, i.e., through the relation

(A95) $$\begin{array}{cc} j=\pi_{V}\left(k\right) & \;⟺\;U^{\left(j\right)}\;\mathrm{is}\;\mathrm{equivalent}\;\mathrm{to}\;\omega \left(V,\cdot \right)\;U^{(k),}\\ j=\pi_{W}\left(k\right) & \;⟺\;U^{\left(j\right)}\;\mathrm{is}\;\mathrm{equivalent}\;\mathrm{to}\;\omega \left(W,\cdot \right)\;U^{\left(k\right)}\;. \end{array}$$

Now, the element VW is associated with the permutation $\pi_{V}\circ \pi_{W}$, while the element WV is associated with the permutation $\pi_{W}\circ \pi_{V}$. On the other hand, the characters obey the equality

(A96) $$\begin{array}{c} \omega (VW,g)=\omega (V,g)\omega (W,g)=\omega (WV,g)\;\forall g\in G\;. \end{array}$$

Hence, we conclude that $\pi_{V}\circ \pi_{W}$ and $\pi_{W}\circ \pi_{V}$ are, in fact, the same permutation. Hence, the elements of the adversarial group must correspond to an Abelian subgroup of the permutations of Irr(U). ☐

Combining Lemmas A19 and A20, we can now prove Theorem 3.

**Proof of Theorem 3.** For different permutations in A, we can choose the isomorphisms $S_{\pi (k),k}:M_{k}\to M_{\pi (k)}$ such that the following property holds:

(A97) $$\begin{array}{c} S_{\pi_{2}\circ \pi_{2}\left(k\right),k}=S_{\pi_{2}\left(\pi_{1}\left(k\right)\right),\pi_{1}\left(k\right)}\;S_{\pi_{1}\left(k\right),k}\;,\;\forall \pi_{1},\pi_{2}\;\in A\;. \end{array}$$

When this is done, the unitary operators $U_{\pi }$ defined in Equation (A93) form a faithful representation of the Abelian group A. Using the canonical decomposition of Theorem A19, every element of $V\in G_{B}$ is decomposed *uniquely* as $V=U_{\pi }\;V_{0}$, where $V_{0}$ is an element of the commutant $U^{\prime }$. Note also that the commutant $U^{\prime }$ is a normal subgroup of the adversarial group: indeed, for every element $V\in G_{B}$ we have $VU^{\prime }V^{†}=U^{\prime }$. Since $U^{\prime }$ is a normal subgroup and the decomposition $V=U_{\pi }V_{0}$ is unique for every $V\in G_{B}$, it follows that the adversarial group $G_{B}$ is the semidirect product $A⋉U^{\prime }$. ☐

## Appendix J. Example: The Phase Flip Group

Consider the Hilbert space $H_{S}=C^{2}$, and suppose that agent *A* can only perform the identity channel and the phase flip channel Z, defined as

(A98) $$\begin{array}{c} Z(\cdot )=Z\cdot Z\;,\;Z=|0\rangle \langle 0|-|1\rangle \langle 1|\;. \end{array}$$

Then, the actions of agent *A* correspond to the unitary representation

(A99) $$\begin{array}{c} U:Z_{2}\to \mathrm{Lin}\left(S\right)\;,\;k\mapsto U_{k}=Z^{k}\;. \end{array}$$

The representation can be decomposed into two irreps, corresponding to the one-dimensional subspaces $H_{0}=\mathrm{Span}\{|0\rangle \}$ and $H_{1}=\mathrm{Span}\{|1\rangle \}$. The corresponding irreps, denoted by

(A100) $$\begin{array}{cc} \omega_{0}:\; & Z_{2}\to C\;,\;\omega \left(k\right)=1,\\ \omega_{1}:\; & Z_{2}\to C\;,\;\omega \left(k\right)={\left(-1\right)}^{k}, \end{array}$$

are the only two irreps of the group and are multiplicative characters.

The condition $VU_{k}=U_{k}V$ yields the solutions

(A101) $$\begin{array}{c} V=e^{i\theta_{0}}\;|0\rangle \langle 0|+e^{i\theta_{1}}\;|1\rangle \langle 1|\;,\;\theta_{0},\theta_{1}\in \left[0,2\pi \right)\;, \end{array}$$

corresponding to the commutant $U^{\prime }$. The condition $VU_{k}={\left(-1\right)}^{k}\;U_{k}V$ yields the solutions

(A102) $$\begin{array}{c} V=e^{i\theta_{0}}\;|0\rangle \langle 1|+e^{i\theta_{1}}\;|1\rangle \langle 0|\;,\;\theta_{0},\theta_{1}\in \left[0,2\pi \right)\;. \end{array}$$

It is easy to see that the adversarial group $G_{B}$ acts irreducibly on $H_{S}$.

Let us consider now the subsystem $S_{A}$. The states of $S_{A}$ are equivalence classes under the relation

(A103) $$\begin{array}{c} |\psi \rangle {≃}_{A}|\psi^{\prime }\rangle \;\exists V\in G_{B}:\;|\psi^{\prime }\rangle =V|\psi \rangle \;. \end{array}$$

It is not hard to see that the equivalence class of the state |ψ〉 is uniquely determined by the *unordered* pair {|〈0|ψ〉|,|〈1|ψ〉|}. In other words, the state space of system $S_{A}$ is

(A104) $$\begin{array}{c} \mathrm{St}\left(S_{A}\right)=\left\{\;\{p,1-p\}\;,:\;p\in [0,1]\right\}\;. \end{array}$$

Note that, in this case, the state space is not a convex set of density matrices. Instead, it is the quotient of the set of diagonal density matrices, under the equivalence relation that two matrices with the same spectrum are equivalent.

Finally, note that the transformations of system $S_{A}$ are trivial: since the adversarial group $G_{B}$ contains the group $G_{A}$, the group $G\left(S_{A}\right)=\pi_{A}\left(G_{A}\right)$ is trivial, namely

(A105) $$\begin{array}{c} G\left(S_{A}\right)=\left\{I_{S_{A}}\right\}\;. \end{array}$$

## Appendix K. Proof of Theorem 4

Let G be a connected Lie group, and let g be the Lie algebra. Since G is connected, the exponential map reaches every element of the group, namely G=exp[ig].

Let h∈G be a generic element of the group, written as h=exp[iX] for some X∈g, and consider the one-parameter subgroup H={exp[iλX],λ∈R}. For a generic element g∈H, the corresponding unitary operator can be expressed as $U_{g}=\exp\left[i\lambda K\right]$, where K∈Lin(S) is a suitable self-adjoint operator. Similarly, the multiplicative character has the form ω(g)=exp[iλμ], for some real number μ∈R.

Now, every element *V* of the adversarial group must satisfy the relation

(A106) $$\begin{array}{c} V\exp\left[i\lambda K\right]=\exp[i\lambda \left(K+\mu \;I_{S}\right)]V\;\forall \lambda \in R\;, \end{array}$$

or equivalently,

(A107) $$\begin{array}{c} \exp\left[i\lambda K\right]=V^{†}\;\exp\left[i\lambda \left(K+\mu \;I_{S}\right)\right]\;V\;\forall \lambda \in R\;. \end{array}$$

Since the operators exp[iλK] and $\exp[i\lambda \left(K+\mu \;I_{S}\right)]$ are unitarily equivalent, they must have the same spectrum. This is only possible if the operators *K* and $K+\mu \;I_{S}$ have the same spectrum, which happens only if μ=0.

Now, recall that the one-parameter Abelian subgroup H is generic. Since every element of G is contained in some one-parameter Abelian subgroup H, we showed that ω(g)=1 for every g∈G.

To conclude the proof, observe that the map $U^{\left(j\right)}\mapsto \omega \;U^{\left(j\right)}$ is the identity, and therefore induces the trivial permutation on the set of irreps Irr(U). Hence, the group of permutations A induced by multiplication by ω contains only the identity element. ☐

## Appendix L. Proof of Proposition 16

**Proof.** It is enough to decompose the two states as

(A108) $$\begin{array}{c} |\psi \rangle =\underset{j\in \mathrm{Irr}(U)}{⨁}\;\sqrt{p_{j}}\;|\psi_{j}\rangle \;\mathrm{and}\;|\psi^{\prime }\rangle =\underset{j\in \mathrm{Irr}(U)}{⨁}\;\sqrt{p_{j}^{\prime }}\;|\psi_{j}^{\prime }\rangle \;, \end{array}$$

where $|\psi_{j}\rangle$ and $|\psi_{j}^{\prime }\rangle$ are unit vectors in $R_{j}⊗M_{j}$. Using this decomposition, we obtain

(A109) $$\begin{array}{c} T_{B}(|\psi \rangle \langle \psi |)=\underset{j\in \mathrm{Irr}(U)}{⨁}\;p_{j}\;\rho_{j}\;\mathrm{and}\;T_{B}(|\psi \rangle \langle \psi |)=\underset{j\in \mathrm{Irr}(U)}{⨁}\;p_{j}^{\prime }\;\rho_{j}^{\prime }\;, \end{array}$$

where $\rho_{j}$ ($\rho_{j}^{\prime }$) is the marginal of $|\psi_{j}\rangle$ ($|\psi_{j}^{\prime }\rangle$) on system $R_{j}$. It is then clear that the equality $T_{B}(|\psi \rangle \langle \psi |)=T_{B}(|\psi^{\prime }\rangle \langle \psi^{\prime }|)$ implies $p_{j}=p_{j}^{\prime }$ and $\rho_{j}=\rho_{j}^{\prime }$ for every *j*. Since the states $|\psi_{j}\rangle$ and $|\psi_{j}^{\prime }\rangle$ have the same marginal on system $R_{j}$, there must exist a unitary operator $U_{j}:M_{j}\to M_{j}$ such that

(A110) $$\begin{array}{c} |\psi_{j}^{\prime }\rangle =\left(I_{R_{j}}⊗U_{j}\right)\;|\psi_{j}\rangle \;. \end{array}$$

We can then define the unitary gate

(A111) $$\begin{array}{c} U_{B}=\underset{j\in \mathrm{Irr}(U)}{⨁}\;\left(I_{R_{j}}⊗U_{j}\right)\;, \end{array}$$

which satisfies the property $U_{B}\left|\psi \rangle =\right|\psi^{\prime }\rangle$. By the characterization of Equation (89), $U_{B}$ is an element of $G_{B}$. ☐
